# Multifunctional hyaluronic acid-based biomimetic/pH-responsive hybrid nanostructured lipid carriers for treating bacterial sepsis

**DOI:** 10.1186/s12929-024-01114-6

**Published:** 2025-02-11

**Authors:** Eman Elhassan, Calvin A. Omolo, Mohammed A. Gafar, Eman A. Ismail, Usri H. Ibrahim, Rene Khan, Mathieu Lesouhaitier, Paul Kubes, Thirumala Govender

**Affiliations:** 1 Discipline of Pharmaceutical Sciences, College of Health Sciences, University of KwaZulu-Natal, Private Bag X54001, Durban, South Africa; 2 Department of Pharmaceutics and Pharmacy Practice, School of Pharmacy and Health Sciences, United States International University-Africa, P. O. Box 14634-00800, Nairobi, Kenya; 3 Department of Pharmaceutics, Faculty of Pharmacy, University of Khartoum, Khartoum, Sudan; 4 Department of Pharmaceutics, Faculty of Pharmacy, University of Gezira, Wad Medani, Sudan; 5 Discipline of Human Physiology, School of Laboratory Medicine and Medical Sciences, College of Health Sciences, University of KwaZulu-Natal, Durban, South Africa; 6 Discipline of Medical Biochemistry, School of Laboratory Medicine and Medical Science, University of KwaZulu-Natal, Durban, South Africa; 7 Department of Physiology and Pharmacology, Cumming School of Medicine, University of Calgary, Calgary, AL Canada

**Keywords:** Biomimetic, PH-responsive, Hyaluronic acid-lysine, Hybrid nanocarriers, Bacterial sepsis, Antibiotic delivery

## Abstract

**Introduction:**

The application of biomimetic and stimuli-responsive nanocarriers displays considerable promise in improving the management of bacterial sepsis and overcoming antimicrobial resistance. Therefore, the study aimed to synthesize a novel hyaluronic acid-lysine conjugate (HA-Lys) and to utilize the attributes of the synthesized HA-Lys with Tocopherol succinate (TS) and Oleylamine (OLA) in the formulation of multifunctional biomimetic pH-responsive HNLCs loaded with vancomycin (VCM-HNLCs), to combat bacterial sepsis.

**Methods:**

A novel hyaluronic acid-lysine conjugate (HA-Lys) was synthesized and characterized using FTIR and ^1^H NMR spectroscopy. Vancomycin-loaded hybrid nanosystems (VCM-HNLCs) were prepared through hot homogenization ultrasonication and evaluated for particle size, polydispersity index (PDI), zeta potential (ZP), and encapsulation efficiency (EE%). In vitro biocompatibility was assessed via MTT assay and red blood cell hemolysis test. The binding affinity to TLR2 and TLR4 was measured using microscale thermophoresis (MST). Drug release was evaluated using the dialysis bag method. Antimicrobial activity against MRSA and efflux pump inhibition were also determined. Efficacy was demonstrated in an MRSA-induced sepsis mice model.

**Results:**

The VCM-HNLCs, produced via hot homogenization ultrasonication, exhibited particle size (PS), polydispersity index (PDI), zeta potential (ZP), and encapsulation efficiency (EE%) of 110.77 ± 1.692 nm, 0.113 ± 0.022, − 2.92 ± 0.210 mV, and 76.27 ± 1.200%, respectively. In vitro*,* biocompatibility was proven by hemolysis and cytotoxicity studies. The VCM-HNLCs demonstrated targetability to human Toll-like receptors (TLR 2 and 4) as validated by microscale thermophoresis (MST). VCM-HNLCs showed a twofold reduction in MIC values at physiological pH compared to the bare VCM against *S. aureus* and MRSA for 48 h. While at pH 6.0, MIC values were reduced by fourfold in the first 24 h and by eightfold in the subsequent 48 and 72 h against tested strains. Furthermore, VCM-HNLCs showed inhibitory effects against MRSA efflux pumps, reactive oxygen species (ROS), and lipopolysaccharide (LPS)-induced hyperinflammation. In an MRSA-induced sepsis mice model, VCM-HNLCs demonstrated superior efficacy compared to free VCM, significantly eliminated bacteria and improved survival rates.

**Conclusions:**

Overall, these results highlight the potential of VCM-HNLCs as novel multifunctional nanocarriers to combat antimicrobial resistance (AMR) and enhance sepsis outcomes.

**Graphical Abstract:**

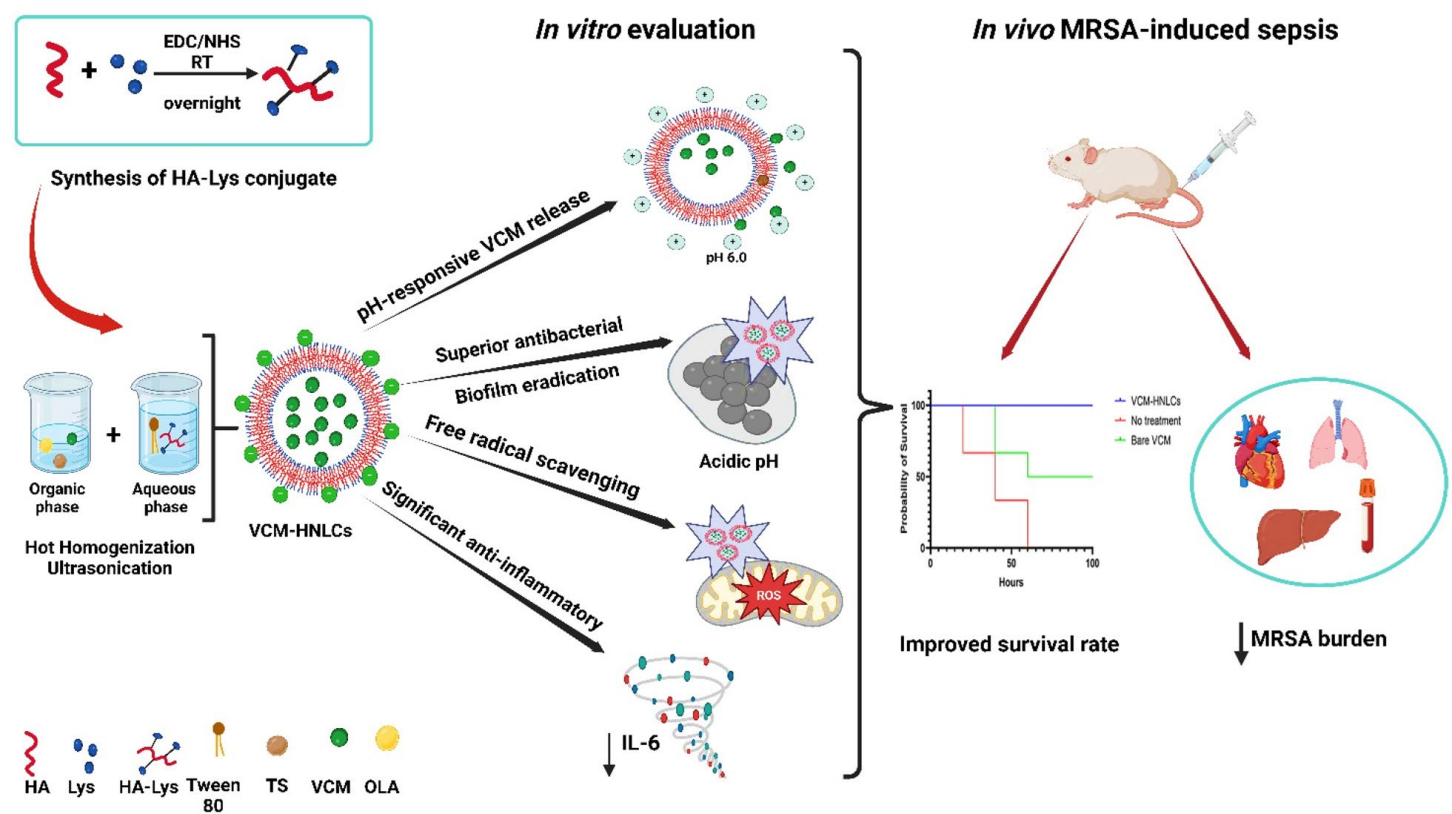

**Supplementary Information:**

The online version contains supplementary material available at 10.1186/s12929-024-01114-6.

## Introduction

Bacterial sepsis, a life-threatening organ dysfunction, arises from a dysregulated host immune response to infection [1]. It poses a significant public health concern globally, with high morbidity and mortality rates despite advances in medical care. According to the Global Burden of Disease Report by the World Health Organization (WHO), sepsis affects more than 49 million people annually, resulting in approximately 11 million deaths, accounting for 20% of all global deaths [2]. Additionally, about 30% of septic patients who survive the initial inflammatory storm may encounter long-term immunosuppressive dysfunctions and cognitive impairment, further burdening individuals and society [3, 4]. The emergence of antimicrobial resistance (AMR) further complicates infection therapy and sepsis management in all settings, especially in high-risk populations, such as infants and patients in intensive care units (ICUs) [2, 5, 6].

The pathophysiology of sepsis involves a complex cascade of events, including the release of pro-inflammatory cytokines, activation of coagulation pathways, overproduction of reactive oxygen species (ROS), endothelial and organ dysfunction, and impaired immune response [7]. In addition, the dysregulated immune response in sepsis can lead to immunosuppression, increasing patients' vulnerability to secondary infections and worsening the clinical course of sepsis [8, 9]. Thus, innovative multi-task therapeutic approaches and enhanced antibiotic treatments are urgently needed to mitigate AMR and control the dysregulated immune response during sepsis.

During infections, pattern recognition receptors in the host cell, such as toll-like receptors (TLRs), are activated by microbial toxins such as peptidoglycan (PGN) of gram-positive bacteria or lipopolysaccharide (LPS) of gram-negative bacteria, enabling the innate immune system to identify invasive pathogens such as bacteria [10]. However, in severe bacterial sepsis, excessive activation of TLRs occurs, leading to systemic inflammation and organ damage [11, 12]. Researchers have identified small drug molecules and antibodies that prevent TLR ligands from binding to the receptor, and they have shown promising results in controlling TLR-induced hyperinflammation in many inflammatory diseases like sepsis [13–18]. Therefore, this biomimetic strategy of minimizing TLR activation by inhibiting their binding to bacterial toxins can be applied to designing a targeted nanobiotic with enhanced bacterial killing potential and modulation of inflammation during sepsis.

Nanostructured lipid carriers (NLCs) have emerged as a promising approach in drug delivery, particularly for treating bacterial infections and related inflammatory disorders [19, 20]. These nanoparticles (NPs) offer numerous advantages, including nanosized-dependent properties, tissue-targeted drug delivery, ease of fabrication, and cost-efficiency, making these delivery systems more attractive [21–24]. Recently, hybrid NLCs-based nanosystems (HNLCs) have been developed by incorporating different polymeric biomaterials into the NLCs make-up to increase their stability, improve their pharmacokinetic properties and enhance the encapsulation of hydrophilic drugs in a lipid-based nanoformulation [25, 26]. Limited studies have reported the use of HNLCs for the delivery of antihypertensive [25], anticancer [27, 28] and antimalarial drugs to date [29]. Only one study reported the application of chitosan-based HNLCs for antibiotic delivery [30]. Therefore, there is a need to explore innovative combinations of materials with multifunctional properties to develop biomimetic pH-responsive HNLCs with enhanced efficacy and targeted antibiotic delivery against bacterial sepsis.

High molecular weight hyaluronic acid (HA) has gained attention as a potential therapeutic agent in managing inflammation due to its well-known anti-inflammatory and immunosuppressive properties [31]. Several studies have reported the ability of HA to reduce the overactivation of TLRs by selectively binding to the receptor and inhibiting their binding to bacterial endotoxins [32–35]. In contrast, L-Lysine (Lys), an essential amino acid, has shown potential in managing oxidative stress related to many disease conditions, including diabetes, chronic kidney disease, lung infections and sepsis [36–38]. This effect plays a beneficial role in scavenging and neutralizing ROS generated during the inflammatory process, thereby reducing tissue damage during sepsis [39]. Combining HA with Lys into a novel HA-Lys conjugate could, therefore, offer a novel therapeutic adjuvant with increased potency against sepsis.

Tocopherol succinate (TS), a vitamin E derivative, has also shown potential in treating various infectious diseases, primarily due to its antioxidant, anti-inflammatory and antimicrobial properties [40, 41]. It has shown promising efflux pump inhibition activities, mainly against NorA and NorB of gram-positive bacteria, allowing for more enhanced intracellular accumulation of antibiotics, making it suitable for combating multidrug-resistant (MDR) bacteria [42–44]. Moreover, it has been reported that TS has significant anti-adhesive properties against *Staphylococcus aureus* (*S. aureus*) biofilms [45, 46]. On the other hand, Oleylamine (OLA), a pH-responsive lipid, offers unique properties that can be exploited in the design of drug delivery systems. Since bacterial infections can lower the physiological pH to acidic levels at the infectious sites, combining the pH-dependent functionality of OLA with its intrinsic antimicrobial behavior could enable the development of lipid-based nanosystems with controlled drug release and improved therapy against infectious diseases [47]. Overall, designing a multifunctional HNLC that combines the benefits of HA-Lys conjugate, TS and OLA could improve the therapeutic index of antibiotics and enhance the performance of the drug delivery system against bacterial sepsis.

Therefore, the study aimed to synthesize a novel HA-Lys conjugate and to utilize the attributes of the synthesized HA-Lys with TS and OLA in the formulation of multifunctional biomimetic pH-responsive HNLCs loaded with vancomycin (VCM-HNLCs), to combat bacterial sepsis. We hypothesize that HA-Lys may directly and competitively bind to host TLR2 or TLR4, preventing their binding to bacterial toxins, reducing excessive TLR activation and neutralizing ROS. Also, TS may potentially reduce bacterial antibiotic resistance via its potential activity against biofilm formation and efflux pumps. In addition, the on-demand release of VCM from the HNLCs could be enhanced through the pH sensitivity of OLA. To our knowledge, this is the first report on synthesizing and utilization of a modified HA-Lys polymer as a targeting adjuvant to treat bacterial sepsis. Also, this study reports the first stimuli-responsive HNLCs for the delivery of any class of drug and the first combined biomimetic and pH-responsive HNLCs to deliver antibiotics against bacterial sepsis. The data obtained from the synthesis and characterization of HA-Lys conjugate, formulation and optimization of VCM-HNLCs, in vitro characterization and in vitro antibacterial, antioxidant and anti-inflammatory studies are provided herein.

## Materials and methods

### Materials

L-Lysine (Lys), tocopherol succinate (TS), Oleylamine (OLA), 1-ethyl-3-(dimethyl amino propyl) carbodiimide (EDC), N-hydroxy succinimide (NHS), ethidium bromide, peptidoglycan (PGN) from *Staphylococcus aureus*, lipopolysaccharides (LPS) from *Escherichia coli* (O55:B5) and 2,2-diphenyl-1-picrylhydrazyl (DPPH) were purchased from Sigma-Aldrich (USA). Hyaluronic acid sodium salt (average molecular weight = 10 kDa), *Staphylococcus aureus* (ATCC 25923) and Multi-drug resistant *Staphylococcus aureus* (SA Rosenbach ATCC BAA 70699) were obtained from DLD Scientific (South Africa). Vancomycin hydrochloride was procured from Sino-bright Import and Export Co., Ltd (China) and then converted to vancomycin free-base (VCM) using a previously described method [48]. Dulbecco’s modified Eagle’s medium (DMEM), Eagle’s Minimum Essential Medium (EMEM), fetal bovine serum, propidium iodide (PI) and Syto9 were obtained from Thermo Fisher (USA). 3(4,5-Dimethylthiazol-2-yl)−2,5-diphenyltetrazolium bromide (MTT), Tween 80, dimethyl sulfoxide (DMSO) and crystal violet were purchased from Merck Chemicals (Germany). Cell lines (HEK 293 - ATCC CRL-1573 and Hep G2-ATCC HB-8065)] were purchased from Highveld Biologicals (Johannesburg, South Africa) and cell culture materials were purchased from Whitehead Scientific (Johannesburg, South Africa). Sheep blood used for hemolytic activity evaluations was obtained from United Scientific (South Africa). Mueller–Hinton Broth (MHB) and Mueller–Hinton Agar (MHA) were obtained from Biolab (South Africa). Recombinant human TLR2 (RPA663Hu03) was purchased from Biocom Africa (Centurion, South Africa). Recombinant human TLR4 (Active ab233665) was obtained from Abcam (Cambridge, UK). Enzyme-linked Immunosorbent Assay (ELISA) kits were purchased from ABclonal Technology (Wuhan, Hubie, China). Microscale thermophoresis (MST) reagents were purchased from Nano Temper Technologies (Germany). A mill-Q water purification system (Millipore Corp., USA) was used to obtain purified milli-Q water. All solvents and reagents used in this study were of analytical grade.

### Synthesis and characterization of HA-Lys conjugate

**Scheme 1.**
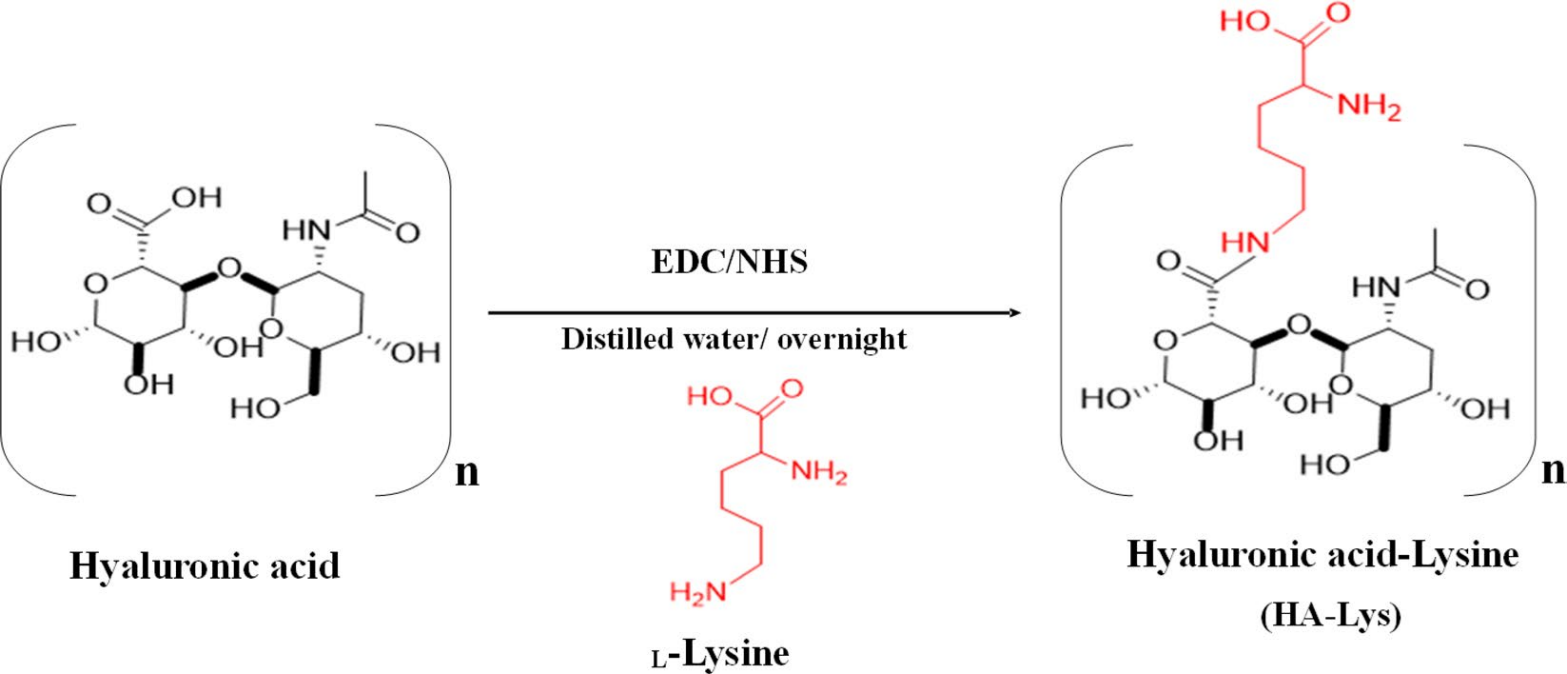
Synthesis of HA-Lys conjugate using a one-step amidation reaction. (EDC: 1-ethyl-3- (dimethyl aminopropyl) carbodiimide, NHS: *N*-hydroxy succinimide)

The HA-Lys conjugate was synthesized by forming an amide bond between the primary amino group of Lys and the free carboxylic acid group of HA according to a previously documented EDC/NHS method with minor modifications (Scheme 1) [49, 50]. To activate the carboxyl acid groups of HA, 200 mg (0.496 mmol) of HA was dissolved in 50 mL of deionized water. Next, EDC (479.75 mg, 2.5 mmol) and NHS (287.7 mg, 2.5 mmol) were added to the HA solution, and the mixture was continuously stirred at room temperature for 15 min. Afterwards, the reaction mixture was mixed with 250 mg of Lys (1.710 mmol), and it was continuously stirred at room temperature overnight. The resultant conjugate solution was purified by three rounds of dialysis against 0.1% w/v NaCl solution and finally against distilled water, followed by lyophilization at − 40 °C to obtain a white powder with a quantitative yield of 81.12%. The freeze-dried samples were characterized using FT-IR at 4 cm^−1^ resolution and 24 scans averaged (Bruker ALPHA II compact FT-IR spectrometer, UK) and ^1^H NMR (Bruker Advance 400 MHz spectrometer, UK), and then stored at 4 °C for further use. The characterization was as follows: ^1^H NMR: (400 MHz, D_2_O) δ(ppm): HA: δ 1.90 (c), 3.30–3.59 (f, g), and 4.44 (h); Lys: δ 1.18 (a), 1.59–2.30 (b, d), and 2.76 (e) (Figure S1), and FT-IR: 3400, 3345.08, 3301.47, 2921.06, 2887.12, 1628.97, 1576.07, 1507, 1400.24, 1388.44, 1035.89 and 656.64 cm^−1^ (Figure S2).

### Preparation of the hybrid nanoparticles

The VCM-loaded HNLCs (VCM-HNLCs) were formulated using a previously reported hot homogenization/ultrasonication technique with a slight modification [20]. The following method is widely used for other similar compounds [51, 52] and was used to encapsulate vancomycin. TS, OLA, and VCM free base for the lipid phase were dissolved in 2 mL of ethanol and heated at 80 °C for 20 min. The aqueous phase was created by dissolving HA-Lys conjugate and a suitable surfactant in 20 mL of milli-Q water, heated separately at 80 °C for 20 min. Both phases were mixed and homogenized using an Ultra Turrax T-25 homogenizer (IKA Labortechnik, Germany) at 6000 rpm for 15 min at the same temperature. The resulting emulsion was promptly cooled in an ice bath for 30 min after further sonication at 30% amplitude for 10 min using a probe sonicator (Omni Sonic-Ruptor 400 Ultrasonic Homogenizer, USA). Prepared VCM-HNLCs were stirred overnight to remove the organic solvent, with milli-Q water used to adjust the final volume to 20 mL if necessary. Blank (VCM-free) HNLCs were also prepared using the same procedure. The prepared nanoformulations were stored in 4 °C for further characterization. Various surfactants (Span 80, PEG 400, PVP, PLX 188, and Tween 80) were screened to ensure nanosystem stability. Subsequently, various parameters, including lipid: surfactant, drug: lipid and lipid: polymer mass ratios, were evaluated. The optimal formulation, characterized by minimum particle size, polydispersity index (PDI), maximum entrapment efficiency percent (EE%), and drug loading capacity (DL% w/w), was selected for further studies. Each batch was prepared in triplicate.

### Physicochemical characterization of VCM-HNLCs

#### Determination of particle size, polydispersity index (PDI), zeta potential (ZP) and surface morphology

The mean particle size, PDI and ZP of the fabricated VCM-HNLCs were measured using the dynamic light scattering (DLS) method with a Zetasizer Nano ZS90 (Malvern Instruments Ltd., UK) fitted with a 633 nm laser at 173° detection optics. The samples were appropriately diluted in distilled water, and the measurement was carried out in triplicate at 25 °C.

Transmission electron microscopy (TEM) was used to analyze the morphological structure of VCM-HNLCs. On a coated grid surface, a drop of properly diluted VCM-HNLCs formulation was placed. It was then let to dry at room temperature, stained for 30 s with 2% phosphotungstic acid and then visualized at an accelerating voltage of 75 kV to obtain TEM images.

#### Determination of entrapment efficiency percent (EE %) and drug loading capacity (DL % w/w)

A previously described ultrafiltration method was employed to determine the entrapment efficiency (EE %) and the drug loading capacity (DL % w/w) of VCM within the HNLCs [20]. Concisely, 2 mL of the formulation was added to an AmiconUltra-4 centrifugal filter tube (Millipore Corp., USA) with a molecular weight cutoff of 10 kDa and centrifuged at 3000 rpm for 30 min at room temperature. The concentration of free VCM in the supernatant was measured by high-performance liquid chromatography (HPLC) using an LC-2050C- 3D (Shimadzu HPLC, Japan) with UV detection at 280 nm, 0.1% aqueous trifluoracetic acid: acetonitrile (15:85) as mobile phases, Gemini® 5 µm NX-C18 110 Å (250 × 4.6 mm) column, 1 mL/min low-pressure gradient flow rate, 100 μL injection volume, and 10 min run time, with a linear equation of Y = 18,420 (x) – 4555.5 and linearity coefficient (R^2^) of 0.9998 (Figure S3). The experiment was carried out in triplicate, and the EE % and DL % w/w were calculated using the following equations [53].

1 $$\text{EE} \%=\frac{\text{Weight of VCM in the HNLCs}}{\text{Total weight of VCM added}}\times 100$$

2 $$\text{DL} \%=\frac{\text{Weight of VCM in the HNLCs}}{\text{Total weight of NPs}}\times 100$$

#### Thermal profiles

Using a Shimadzu DSC-60 (Japan), thermal analysis was performed to determine the thermograms of HA, Lys, HA-Lys conjugate, TS, VCM, physical mixture and lyophilized VCM-HNLCs. Briefly, 5 mg of each sample was loaded in an aluminum pan, which was then sealed with a crimper and heated under a stream of nitrogen to 330 °C at a steady 1 0 °C/min. An empty pan served as a reference.

### In vitro biosafety studies

#### Cytotoxicity and cell viability assay

The cytocompatibility of the synthesized HA-Lys conjugate and its nanoformulation (VCM-HNLCs) was assessed using human embryonic kidney 293 (HEK 293) and human hepatocyte carcinoma (HepG2) cells, employing a previously reported MTT assay [54, 55]. Briefly, cells were seeded (2.5 × 10^3^ cells/well) in a 96-well plate using 100 μL of DMEM, 10% fetal bovine serum (FBS), 1% pen-strep-fungizone and 1% L-glutamine and incubated at 37 °C for 24 h using a CO_2_ humidified incubator. Subsequently, the cells were incubated for the next 24 h after being exposed to HA-Lys and VCM-HNLCs (diluted with DMEM) at doses of 20, 40, 60, 80, and 100 μg/mL, respectively. After that, the cells were grown for an extra 4 h using a new culture medium (100 μL/well) and 20 μL/well of MTT solution (5 mg/mL in PBS). After removing the supernatant, 100 μL of sterile dimethyl sulfoxide (DMSO) was used to solubilize the formazan crystals. The absorbance at 540 nm was then recorded after 1 h using a microplate spectrophotometer (Spectrostar Nano, Germany). The experiment was carried out in triplicate and the cell viability percent was computed using Eq. (3):

3 $$\text{Cell viability }\%=\frac{\text{A}540\text{ nm treated cells}}{\text{A}540\text{ nm untreated cells}}\times 100$$

#### Hemolysis

The non-hemolytic activity of the prepared VCM-HNLCs was assessed using a previously reported method [56, 57]. In summary, three washes of freshly collected sheep blood were performed using 0.1N sterile isotonic PBS (pH 7.4) by centrifugation at 3000 rpm for 10 min. Next, 1.8 mL of six distinct concentrations of VCM-HNLCs formulation in 0.1N sterile PBS (0.05, 0.1, 0.2, 0.3, 0.4 and 0.5 mg/mL), PBS (as negative control) and distilled water (as positive control) were separately mixed and incubated with 0.2 mL of the previously purified red blood cell suspension for 30 min at 37 °C. After incubation, the samples were centrifuged using the ultracentrifuge (Centurion Scientific, UK) at 3000 rpm and 4 °C for 10 min. The supernatants of each sample were then collected into 96-well plates and their hemoglobin absorbances were quantitatively recorded at 540 nm using a microplate reader (SpectraMax M2, USA). The experiment was done in triplicate and the percentage of non-hemolytic activity was calculated using the following equation:

4 $$\text{Non}-\text{hemolytic activity }\%= 100-\frac{(\text{ABS}-{\text{ABS}}_{0})}{({\text{ABS}}_{100}-{\text{ABS}}_{0})}$$

where ABS_,_ ABS_0_ and ABS_100_ are the absorbances of the sample, positive and negative controls, respectively.

### In vitro binding affinity study

The binding affinity of individual adjuvants (HA, Lys and HA-Lys conjugate), non-hybrid NLCs components and VCM-HNLCs to human TLR2 and TLR4 was determined and compared to their natural substrates, peptidoglycan (PGN) and lipopolysaccharide (LPS), respectively, employing microscale thermophoresis (MST) on a Nano Temper Monolith NT.115 instrument (Nano Temper Technology, Germany) [58].

#### Determination of in vitro binding affinity to TLR2

According to the manufacturer's protocol, recombinant human TLR2 was first adjusted to a concentration of 200 nM, labeled with the Monolith His-Tag labeling kit Red -tris-NTA, incubated for 30 min at room temperature and then centrifuged at 15,000 rpm for 10 min at 4 °C. After protein labeling, serial dilutions of different ligands with varying starting concentrations were prepared using an appropriate MST buffer (0.1 M PBS, 0.05% Tween 20, pH 7.4). Then, each dilution was mixed with an equivalent volume (10 µL) of the labeled TLR2 in a final concentration of 50 nM and the mixture was incubated for 10 min. Samples were then loaded into Standard Treated MST glass capillaries (Nano Temper Technology, Germany) and MST traces were measured using the Nano Temper Monolith NT.115 instrument at 25 °C and 70% MST medium power. All experiments were performed in triplicate. The dissociation constant (K_d_) for each ligand was adjusted using the Monolith-Affinity analysis program version 2.1.3 (Nano Temper Technologies, Germany).

#### Determination of in vitro binding affinity to TLR4

In the experiment, the concentration of recombinant human TLR4 was first adjusted to 3.6 µM and labeled with the Monolith NT.115 protein labeling kit Blue-NHS according to the manufacturer's protocol using a 1:3 protein-to-dye ratio. The mixture was incubated at room temperature for 15 min, protected from light, and then purified with a gel-filtration column. Following protein labeling, serial dilutions of various ligands were prepared using MST buffer (0.1 M PBS, 0.05% Tween 20, pH 7.4) with different starting concentrations. Next, each dilution was supplemented with an equivalent volume of the labeled TLR4 (10 µL) at a final concentration of 20 nM and incubated for 10 min. Finally, the samples were loaded into Standard Treated glass MST capillaries (Nano Temper Technology, Germany) and MST traces were recorded in Nano Temper Monolith NT.115 instrument at 25 °C, blue LED and 80% MST power. All experiments were performed in triplicate. For every tested ligand, the dissociation constant (Kd) values were calculated using Monolith-Affinity analysis software version 2.1.3 (Nano Temper Technologies, Germany).

### In vitro drug release study and linear kinetics

The release behavior of VCM from the VCM-HNLCs in response to different pH values (7.4 and 6.0) was evaluated using a dialysis bag method [58]. Briefly, 2 mL of pure VCM solution (1 mg/mL) and VCM-HNLCs solution (containing 1 mg/mL VCM) were separately placed in cellulose dialysis bags (molecular weight:14,400 Da) and the bags were shaken and dialyzed against 40 mL of the buffer of choice receiver solution (PBS pH 7.4 or 6.0) at 37 °C and 100 rpm. At predetermined intervals, 2 mL samples were withdrawn from the receiver solution and promptly replaced with an equivalent amount of fresh appropriate buffer (PBS pH 7.4 or 6.0) to maintain sink condition. The amount of VCM released per time was determined by HPLC according to the same analysis method mentioned for EE % evaluation (2.4.2). The experiment was performed in triplicate and the cumulative release percentage of VCM was calculated using the following equation:

5 $$\text{Cumulative release }\%=\frac{{Q}_{t}}{{Q}_{v}}\times 100$$

where Qt is the amount of VCM released at time t, and Qv is the total amount of VCM previously used in the VCM-HNLCs formulation.

Different mathematical models, including zero-order, first-order, Higuchi, Krosmeyer-Peppas, Hixson-Crowell and Weibull, were then employed to examine the kinetics of VCM release from the VCM-HNLCs using the software program DDSolver [59]. The best-fit model was selected based on the correlation coefficient (R^2^) and the root mean square error (RMSE), and the release mechanism was identified using Weibull and Krosmeyer-Peppas models by computing the values of the Weibull exponent parameter (β) and the release exponent (n), respectively.

### Short-term physical stability study

The short-term colloidal stability of the prepared VCM-HNLCs formulation was evaluated at two different conditions: room temperature (25 °C) and cold temperature (4 °C) over 90 days of storage. Using a Zetasizer Nano ZS90 (Malvern Instruments Ltd., UK) equipped with a 633 nm laser with detection optics of 173°, the mean particle size, PDI and ZP were measured at 0, 30, 60 and 90 days. All experiments were run in triplicate.

### In vitro antibacterial activity studies

#### Determination of minimum inhibitory concentration (MIC) and fractional inhibitory concentration (FIC)

The antibacterial activity (MIC) of bare VCM, blank HNLCs and VCM-HNLCs against *S. aureus* and MRSA at pH 7.4 and 6.0 was investigated using a broth microdilution method, as previously reported [60]. In brief, *S. aureus* and MRSA cultures in Mueller- Hinton broth (MHB) were grown overnight at 37 °C and 100 rpm using a shaking incubator (Labcon, USA). The bacterial suspensions were adjusted to 0.5 McFarland standard (1.5 × 10^8^ colony-forming units (CFU)/mL) using a DEN-1B densitometer (Latvia) and further diluted to a final concentration of 5 × 10^5^ CFU/mL. Then, 135 µL of MHB solution was pipetted into 96-well plates, and the treatments (bare VCM, blank HNLCs and VCM-HNLCs solutions) were added individually and serially diluted to obtain a range of concentrations. Thereafter, 15 µL of the bacterial suspension was added to each well, and the mixture was incubated for 72 h in the shaking incubator (Labcon, USA) adjusted at 37 °C and 100 rpm. Following incubation, 5 µL of the sample mixtures were spotted on Mueller–Hinton agar (MHA) plates at specified time intervals (24, 48, and 72 h). The MIC values were calculated after 24 h of incubation at 37 °C. All experiments were performed in triplicate.

Based on the MIC values obtained from the aforementioned antibacterial activity study, the cumulative FIC was determined to understand the combined antibacterial activity of the blank HNLCs and VCM in the VCM-HNLCs against *S. aureus* and MRSA at pH 7.4 and 6.0. Equations (6) and (7) were used to calculate the ΣFIC and the FIC index is shown in Table S1.

6 $$\text{FIC }\left({\text{VCM}}\right) = \frac{\text{ (the MIC of }{\text{VCM}}\text{ in combination with HNLCs) }}{\text{(the MIC of VCM)}}$$

7 $$\text{FIC }\left(\text{Blank }{\text{HNLCs}}\right) = \frac{\text{ (the MIC of }{\text{HNLCs}}\text{ in combination with VCM) }}{\text{(the MIC of Blank }{\text{HNLCs}}\text{)}}$$

$$\Sigma \text{FIC}=\text{FIC }(\text{VCM})+\text{FIC }(\text{Blank HNLCs})$$

#### Flow cytometry

The cell viability of MRSA in the population following the treatment with VCM and VCM-HNLCs for 18 h was assessed using a flow cytometry method [61]. To prepare the bacterial cultures, MRSA was grown overnight at 37 °C and 100 rpm in a shaking incubator to a concentration of 0.5 McFarland standard (1.5 × 10^8^ CFU/mL) in MHB. The grown bacteria were then collected by centrifugation at 3000 rpm for 30 min and the supernatant was discarded. The pellets were resuspended in PBS and incubated for an additional 4 h at 37 °C. Afterward, 15 µL of bacterial suspension at a concentration of 5 × 10^5^ CFU/mL was added to a 96-well plate, each containing 135 μL of bare VCM and VCM-HNLCs formulations at the MIC concentrations of 7.8 μg/mL and 3.9 μg/mL, respectively. The sample mixture was further incubated at 37 °C in a shaking incubator (100 rpm) for 18 h. Following incubation, 50 μL of each sample was transferred to the flow cytometry tubes containing 350 μL of sheath fluid and vortexed for 5 min. Then, 5 μL of membrane-impermeable propidium iodide (PI) and membrane-penetrating Syto9 dyes were added to each tube and the mixture was incubated for 30 min in the dark. Samples were analyzed on the flow cytometry device (BECKMAN COULTER DxFLEX, Nyon 1, USA), and at least 10,000 cells were collected in triplicate for each sample. Untreated MRSA cells were used as a negative control.

#### Determination of in vitro antibiofilm activity

##### Crystal violet assay

The microtiter dish biofilm formation assay was employed to evaluate the ability of VCM-HNLCs to remove MRSA biofilms [62, 63]. Bacterial suspension in MHB at pH 7.4 and 6.0 (1.5 × 10^8^ CFU/mL) was added to 96-well plates (200 μL/well) and incubated at 37 °C for 7 days to create mature biofilms. Following incubation, the medium was removed from the wells and non-adherent bacterial cells were eliminated by washing the wells three times with a sterile PBS (pH 7.4 and 6.0). Thereafter, 200 μL of bare VCM solution and VCM-HNLCs formulation at a concentration of 10 × MIC were added to the wells and incubated at 37 °C for 24 h. The excess treatments and unattached bacteria were then removed by washing the wells three times in sterile PBS (pH 7.4 and 6.0), followed by staining of the biofilms with 0.01% w/v crystal violet solution for 15 min. Later, the excess dye was gently aspirated, thoroughly washed using PBS pH 7.4 and 6.0, and left to dry overnight. To elute the crystal violet stain, 30% acetic acid solution (125 μL) was added to each well and incubated for 10 min. The eluted dye was then transferred to another 96-well plate and imaged for qualitative analysis. Subsequently, the intensity of the crystal violet dye was quantitatively measured at 570 nm using a microplate reader (SpectraMax M2, USA) to investigate the antibiofilm effect of the test samples.

##### Florescence microscope assay

To confirm the ability of VCM-HNLCs to eliminate MRSA biofilms, a florescence microscope assay was conducted using glass microscope coverslips (18 mm × 18 mm) as carriers for biofilm formation. [61]. Coverslips were initially placed at the bottom of 6-well plates, and then 2 mL of bacterial suspension in MHB (1.5 × 10^8^ CFU/mL) was added to each well. The plates were incubated for 7 days at 37 °C to allow biofilms to grow over the coverslips. After developing mature biofilms, non-adherent bacterial cells were removed from the wells by washing them three times with PBS pH 7.4 using a Pasteur pipette. The wells containing biofilms were then treated with 2 mL of PBS pH 7.4 (as a control) and 2 mL of bare VCM and VCM-HNLCs solutions (5 × MIC) and incubated for 12 h at 37 °C. The treatments and unattached bacteria were removed from the wells by washing them twice with PBS pH 7.4. After that, the coverslips were stained with a solution of Syto9 and propidium iodide (PI) (30 μL diluted with 1 mL of distilled water) for 30 min in the dark to avoid photobleaching. After the plates were washed with PBS pH 7.4 to remove excess dye, the coverslips were inverted on a glass microscope slide, and the edges were gently glued. Fluorescence microscope images were captured and processed using Nikon Eclipse 80i FM (Japan) to visualize the biofilm eradication effects of bare VCM and VCM-HNLCs.

#### Time-killing assay (TKA)

The time-killing kinetics of the prepared VCM-HNLCs against MRSA were evaluated at pH 7.4 and 6.0 using a previously described colony counting method [64]. In brief, an overnight MRSA culture (0.5 McFarland standard) was diluted into concentrations of 10^5^–10^6^ CFU/mL using sterile PBS (pH 7.4 and 6.0). Then, bare VCM and VCM-HNLCs solutions at 5 × MIC were added to the diluted bacteria and incubated in the shaking incubator (Labcon, USA) at 100 rpm and 37 °C. Untreated bacteria in PBS solution (pH 7.4 and 6.0) was used as a control. At specified time intervals (0, 1, 2, 4, 6, 8, 12 and 24 h), 0.2 mL of each sample was withdrawn and subjected to serial dilution with sterile PBS (pH 7.4 and 6.0). Subsequently, 0.1 mL of each diluted sample was sub-cultured on an MHA plate and incubated for 24 h. After incubation, the number of colonies formed in each dilution plate was counted and converted into CFU/mL using Eq. (8). The TKA of the prepared samples was determined by plotting log_10_ CFU/mL versus time. The experiment was performed in triplicate.

8 $$\text{CFU/m}{\text{L}}\text{ } = \frac{\text{ no. of colonies }\times \, \text{dilution factor} \, }{\text{The v}\text{olume of dilution taken}}$$

#### Efflux pump inhibition

The potential of VCM-HNLCs to suppress MRSA efflux pumps was evaluated using a simple cartwheel method according to previously reported protocols with minor modifications [65, 66]. Briefly, MHA plates containing 2 µg/mL ethidium bromide (EtBr) were freshly prepared and protected from light on the same day of the experiment. An overnight culture of MRSA (0.5 McFarland standard) was seeded into a 96-well plate and treated with bare VCM and VCM-HNLCs formulation at a concentration of 0.25 × MIC values. A group of untreated bacteria was served as a control. The samples were incubated at 37 °C for 1 h and then streaked on the MHA containing EtBr in a cartwheel form. All plates were incubated in the dark at 37 °C for 24 h. The plates were then visualized using a UV trans-illuminator (Uvitec Cambridge, UK) to observe any residual fluorescence exhibited by the bacteria. The inhibitory effect of the tested samples on the MRSA efflux pumps can be identified from the fluorescence emission under the UV transilluminator.

### In vitro antioxidant study

Following a previously described DPPH radical scavenging method with minor modifications, the antioxidant activity of TS, Lys, HA-Lys and VCM-HNLCs through free radical scavenging was investigated [67]. A methanolic solution of DPPH at a concentration of 0.5 mM and various concentrations of the treatments in distilled water (20–1000 µg/mL) were individually prepared. Subsequently, 150 µL of each formulation sample was mixed with an equal volume of DPPH solution in a 96-well plate and left to incubate at 37 °C in the dark. A negative control consisting of a mixture of DPPH and water (150 µL each) was used. The absorbance of the reaction mixture was measured at 517 nm after 1 h of incubation using a microplate reader (SpectraMax M2, USA). The assay was performed in triplicate and ascorbic acid was used as a positive control. The following equation was used to compute the percentage of antioxidant activity:

9 $$\text{Antioxidant }\%=\frac{\text{A}516\text{ nm control}-\text{A}516\text{ nm sample}}{\text{A}516\text{ nm control}}\times 100$$

### In vitro anti-inflammatory studies

#### Cytoprotective effect against LPS-induced cytotoxicity

The cytoprotective effect of HA-Lys and VCM-HNLCs against LPS-induced cytotoxicity was assessed in vitro using previously documented methods utilizing an MTT assay [55, 68, 69]. In brief, HepG2 cells were cultured in DMEM supplemented with glutamine, antibiotics, and fetal bovine serum and incubated at 37 °C in a humidified atmosphere containing 5% carbon dioxide. When the cells reached 80% confluency, they were plated in a 96-well plate (20,000 cells/well) and left for 24 h. The cells were then subjected to a new set of treatments for a further 24 h, which included: (a) 200 µL of DMEM was used as the control; (b) 200 µL of 0.9 mg/mL of LPS was used as the LPS treatment; (c) 200 µL of 0.9 mg/mL of LPS and 100 µg/mL of HA-Lys were used as the HA-Lys treatment; (d) 200 µL of 0.9 mg/mL of LPS and 100 µg/mL of VCM-HNLCs in DMEM were used as the nanoformulation treatment. Following a 24 h, the media were removed and replaced with 100 µL/well of fresh DMEM mixed with 20 µL/well of 5 mg/mL MTT solution. 4 h later, the media was replaced with 100 µL/well sterile DMSO to solubilize the formazan crystals. Finally, absorbance from each well was measured at 570 nm using a microplate spectrophotometer (Spectrostar Nano, Germany) after 1 h of DMSO addition. Each treatment was carried out in triplicate, and the percentage of viable cells was determined.

#### Anti-inflammatory activity against LPS-induced inflammatory responses (measurement of IL-6 levels)

The anti-inflammatory activity of HA-Lys and VCM-HNLCs was further examined using an LPS-stimulated HepG2 cells model, as previously described [68]. In this study, IL-6 levels were measured using Enzyme-Linked Immunosorbent Assay (ELISA) kits following the stimulation of HepG2 cells with 1 µg/mL LPS solution in DMEM for 12 h, either with or without treatments. After HepG2 cells were cultured and plated in the same manner described in the previous section (Cytoprotective effect against LPS-induced cytotoxicity), they were subjected to the following treatments for a duration of 12 h: (a) Control untreated (200 µL of DMEM); (b) LPS-treated (200 µL of 1 µg/mL of LPS in DMEM); (c) HA-Lys-treated (200 µL of 1 µg/mL of LPS and 50 µg/mL or 100 µg/mL HA-Lys in DMEM); (d) VCM-HNLCs-treated (200 µL of 1 µg/mL of LPS and 50 µg/mL or 100 µg/mL VCM-HNLCs in DMEM). After that, the media were collected to determine the amount of IL-6 in the supernatants using ELISA kits following the manufacturer’s instructions.

### In vivo studies

#### Mice

As proof-of-concept, animal experiments were performed with WT C57BL/6 adult mice (6–10 weeks old), and all experimental animal protocols were approved by the University of Calgary Animal Care Committee and were in compliance with the Canadian Council for Animal Care Guidelines. All animals were maintained in a specific pathogen-free environment at the University of Calgary Animal Resource Centre. Mice were housed under standardized temperature (21–22 °C) and illumination (12 h light/12 h darkness) with access to tap water and pelleted food.

#### Staphylococcal strains and culture conditions

*Staphylococcus aureus* strain MW2 used in the in vivo experiments was obtained from NARSA (Network on Antimicrobial Resistance in *Staphylococcus aureus*).

#### Mouse infections and in vivo treatments

*Staphylococcus aureus* strains were subcultured without antibiotics until the exponential phase (OD_660nm_, 1.0), washed and resuspended in saline. Then, 100 μL of 1 × 10^8^ CFUs were injected intravenously (I.V.) into the tail vein for the survival experiment or 5 × 10^7^ CFUs in 200 μl for the bacteria dissemination experiment. Mice were monitored until 100 h after infection for the survival experiment or sacrificed 48 h after infection for the bacteria dissemination assay.

#### Bacteriological analysis

Anesthetized mice were washed with 70% ethanol under sterile conditions. For determination of colony forming units (CFU), 30 μl of tissue homogenate or blood was serially diluted, plated onto brain–heart infusion agar plates, and incubated at 37 °C for 18 h, and then bacterial colonies were counted.

### Statistical analysis

The data were statistically analyzed using GraphPad Prism® version 10 (GraphPad Software Inc., USA), which included paired T-tests and one-way analysis of variance (ANOVA). The difference between compared groups was considered significant if the *P*-value was < 0.05. All results were presented as mean ± standard deviation (SD) and all graphs and figures were generated using GraphPad Prism® 10 and the BioRender website, unless otherwise noted.

## Results

### Synthesis and characterization of HA-Lys conjugate

The Hyaluronic acid-Lysine conjugate (HA-Lys) was successfully synthesized via a one-step amidation reaction, followed by purification through intensive dialysis. Subsequently, the conjugate was characterized using ^1^H NMR and FT-IR [70]. The ^1^H NMR spectrum (Figure S1) clearly showed all the signals attributed to the HA backbone (Figure S1A) δppm 1.90 (CH_3_ groups, c), 3.59 (CH groups in the tetrahydropyran ring, f) and 4.44 (OH groups, g), and to the Lys moieties (Figure S1B) δppm 1.18, 1.59, and 2.76 (CH_2_ groups, a, b and d, respectively), and 3.30 (CH groups, e). Similar signals of both HA and Lys were observed in the ^1^H NMR spectrum of HA-Lys conjugate with slight chemical shifts (Figure S1C). These chemical shifts in particular regions of the ^1^H NMR spectrum of HA-Lys confirmed the conjugation between HA and Lys.

The FT-IR spectrum (Figure S2) revealed a broad stretching band attributed to pure HA (Figure S2A) at 3400.4 cm^−1^ (due to O–H stretching vibration) and 3301.47 cm^−1^ (due to N–H stretching vibration) in the *N*-acetyl side chain. Also, other bands were detected at 2887.12 cm^−1^ (due to symmetric methyl C–H stretching in glucuronic acid side chain), 1628.97 cm^−1^ (due to the amide group of C=O carboxyl), 1338.74 cm^−1^ (due to the C–N stretching of primary aromatic amine) and 1035.89 cm^−1^ (due to C–O alcoholic stretching), respectively. The spectrum also displayed the bands associated with pure lysine (Figure S2B), including those at 3345.08 cm^−1^ (resulting from the overlap of O–H and N–H stretching), 2921.06 cm^−1^ (due to symmetric C–H stretching of the CH2 group), 2129.12 cm^−1^ (from the overtone and combinational bond), 2816 cm^−1^ (resulting from C–H vibration of the CH group), 1576.07 cm^−1^ (due to the in-plane bending of N–H of the free primary amine in Lys), 1507 cm^−1^ (due to asymmetric scissoring of the NH2 group), 1400.24 cm^−1^ (resulting from symmetric vibration of C=O carboxyl and C-N group) and 656.64 cm^−1^ (due to C=O carboxyl rocking), respectively. The FTIR spectrum of HA-Lys conjugate (Figure S2C) exhibited all the characteristic bands of both HA and Lys at the same positions. In addition, a prominent and well-defined band with increased intensity was observed at 1628.59 cm^−1^ (shift from 1628.97), corresponding to the amide group of C=O carboxyl. This intensified band indicates the formation of a new secondary amide bond between the amine group of Lys and the carboxyl group of HA, thus confirming the successful amidation reaction and formation of HA-Lys conjugate.

### Formulation, characterization and optimization of VCM-HNLCs

After validating the structure of the HA-Lys conjugate, its potential for developing HNLCs was explored. Both blank HNLCs and VCM-HNLCs were successfully prepared using the hot homogenization/ultrasonication technique [20]. The initial phase involved assessing various surfactants to determine the most suitable one for producing HNLCs with the desired particle sizes, smaller PDI, and enhanced colloidal stability, as shown in Table S2.

The optimization process involved assessing various formulation parameters using a single factor-at-a-time optimization method. Initially, the lipid: surfactant mass ratio was varied from 1:0.5 to 1:2, with fixed drug: lipid (1:4) and lipid: polymer (1:0.1) ratios. Increasing the lipid: surfactant ratio decreased the particle size from 98.77 to 42.44 nm, while EE% and DL% decreased from 56.18% to 20.78% and from 7.36% to 2.07%, respectively, as indicated in Table 1. Based on this observation, the optimal lipid: surfactant ratio of 1:0.5 was selected for further optimization.

**Table 1:** Size, PDI, ZP, EE% and DL% w/w for the different lipid: surfactant, drug: lipid, and lipid: polymer mass ratios used in the optimization of VCM-HNLCs. Results represented as mean ± SD (n = 3)

Optimization parameter	Size (nm)	PDI	ZP (mV)	EE %	DL % w/w
Lipid: surfactant mass ratio	Fixed drug: lipid (1:4) and lipid: polymer (1:0.1) mass ratios				
1:0.5	98.77 ± 0.436	0.156 ± 0.008	− 2.92 ± 0.332	56.18 ± 3.395	7.36 ± 1.582
1:1	62.29 ± 0.440	0.204 ± 0.007	− 5.41 ± 0.643	35.70 ± 0.375	5.07 ± 0.052
1:2	42.44 ± 1.148	0.233 ± 0.005	− 2.69 ± 0.283	20.78 ± 0.934	2.07 ± 0.093
Drug:lipid mass ratio	Fixed lipid: surfactant (1:0.5) and lipid: polymer (1:0.1) mass ratios				
1:3	90.94 ± 0.0291	0.151 ± 0.011	− 5.59 ± 1.28	45.50 ± 0.024	5.26 ± 0.006
1:4	98.77 ± 0.436	0.156 ± 0.008	− 2.92 ± 0.332	56.18 ± 3.395	7.36 ± 1.582
1:5	99.39 ± 0.485	0.127 ± 0.006	− 2.5 ± 0.064	64.77 ± 0.055	8.28 ± 0.004
Lipid:polymer mass ratio	Fixed lipid: surfactant (1:0.5) and drug: lipid (1:5) mass ratios				
1:0.05	90.58 ± 0.255	0.126 ± 0.004	− 2.54 ± 0.255	55.81 ± 0.448	7.38 ± 0.051
1:0.1	99.39 ± 0.485	0.127 ± 0.006	− 2.5 ± 0.064	64.77 ± 0.055	8.28 ± 0.004
1:0.15	108.0 ± 0.907	0.136 ± 0.009	− 3.16 ± 0.530	72.27 ± 0.280	8.86 ± 0.001

Next, the effect of the drug: lipid ratio was evaluated at a fixed lipid: surfactant ratio of 1:0.5 and lipid: polymer ratio of 1:0.1. Increasing the drug: lipid ratio from 1:3 to 1:5 improved EE% from 45.50% to 64.77% and DL% from 5.26% to 8.28% without significant changes in size, PDI and ZP. Consequently, optimal lipid: surfactant (1:0.5) and drug: lipid (1:5) mass ratios were used to evaluate the effect of different lipid: polymer mass ratios on the properties of VCM-HNLCs.

Lastly, different lipid: polymer ratios were tested, varying from 1:0.05 to 1:0.15, with optimal EE% increasing from 55.81% to 72.27% without significant changes in other parameters. Furthermore, negative ZP values (− 2.5 ± 0.064 to − 5.59 ± 1.28) were observed across all formulations, indicating strong electrostatic repulsion and preventing particle coalescence [71].

Based on these findings, VCM-HNLCs with a lipid: surfactant ratio of 1:0.5, drug: lipid ratio of 1:5, and lipid: polymer ratio of 1:0.1 were identified as the optimal formulation for further studies. These VCM-HNLCs exhibited particle size of 110.77 ± 1.692 nm, PDI of 0.113 ± 0.022, ZP of − 2.92 ± 0.210 mV, EE% of 76.27 ± 1.200%, and DL% of 8.36 ± 0.250%.

**Fig. 1:**
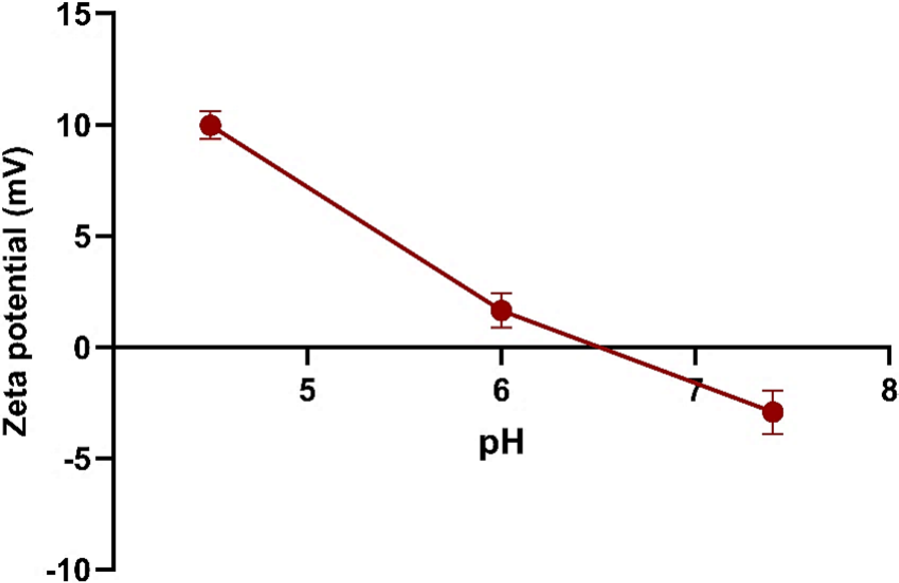
The impact of pH variation on the surface charge of VCM-HNLCs (P*-*value = 0.0004). The data is shown as mean ± SD (n = 3)

The pH responsiveness of the VCM-HNLCs was evaluated, and the formulation demonstrated significant pH-dependent behavior with a shift in charge from negative at pH 7.4 to positive at lower pH values, as depicted in Fig. 1.

### Determination of surface morphology

**Fig. 2:**
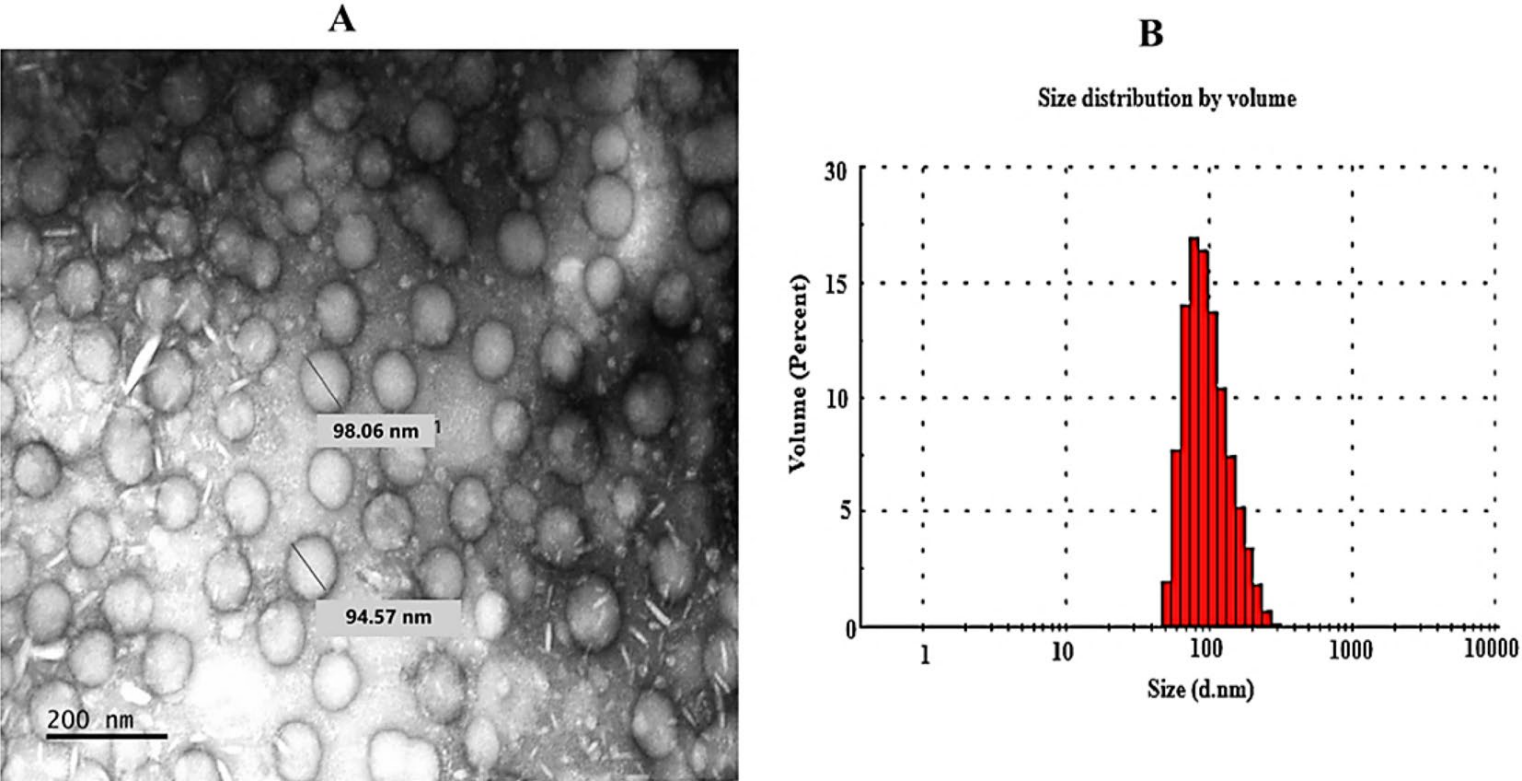
**A** TEM image of VCM-HNLCs and **B** A histogram displaying the size distribution of VCM-HNLCs determined by DLS

Transmission electron microscopy (TEM) was employed to further examine the surface morphology of the prepared VCM-HNLCs. The TEM image of the optimized VCM-HNLCs formulation revealed well-defined spherical-shaped NPs that were consistent in size with those determined by DLS, as illustrated in Fig. 2.

### Thermal profiles

The thermal profiles of individual excipients, the physical mixture, and the lyophilized VCM-HNLCs are presented in Figure S4. The lyophilized VCM-HNLCs were initially reconstituted in DW to evaluate their redissolution capability. The size, PDI, and ZP of the reconstituted nanoparticles were measured using DLS, and the results were comparable to those obtained before lyophilization (size of 113.25 ± 1.342 nm, PDI of 0.119 ± 0.100, ZP of − 2.98 ± 0.150 mV). In the DSC measurements, lysine (Lys), vancomycin (VCM), and Tween 80 (TS) exhibited distinct thermal behaviors, with endothermic peaks observed at 243.5 °C, 109.17 °C, and 83.56 °C, respectively. Hyaluronic acid (HA) displayed an exothermic peak at 233.36 °C. Moreover, the thermograms of the HA-Lys conjugate and the physical mixture of VCM-HNLCs excipients demonstrated similar endothermic and exothermic peaks, albeit with slight shifts. In contrast, the thermogram of the lyophilized formulation exhibited no discernible thermal peaks, indicating a transition from a crystalline to an amorphous state during formulation. Furthermore, the successful entrapment of the VCM in the prepared NPs in an amorphous form was confirmed by the absence of the characteristic VCM peak in the thermogram of the lyophilized VCM-HNLCs.

### In vitro biosafety studies

#### Cytotoxicity and cell viability assay

**Fig. 3:**
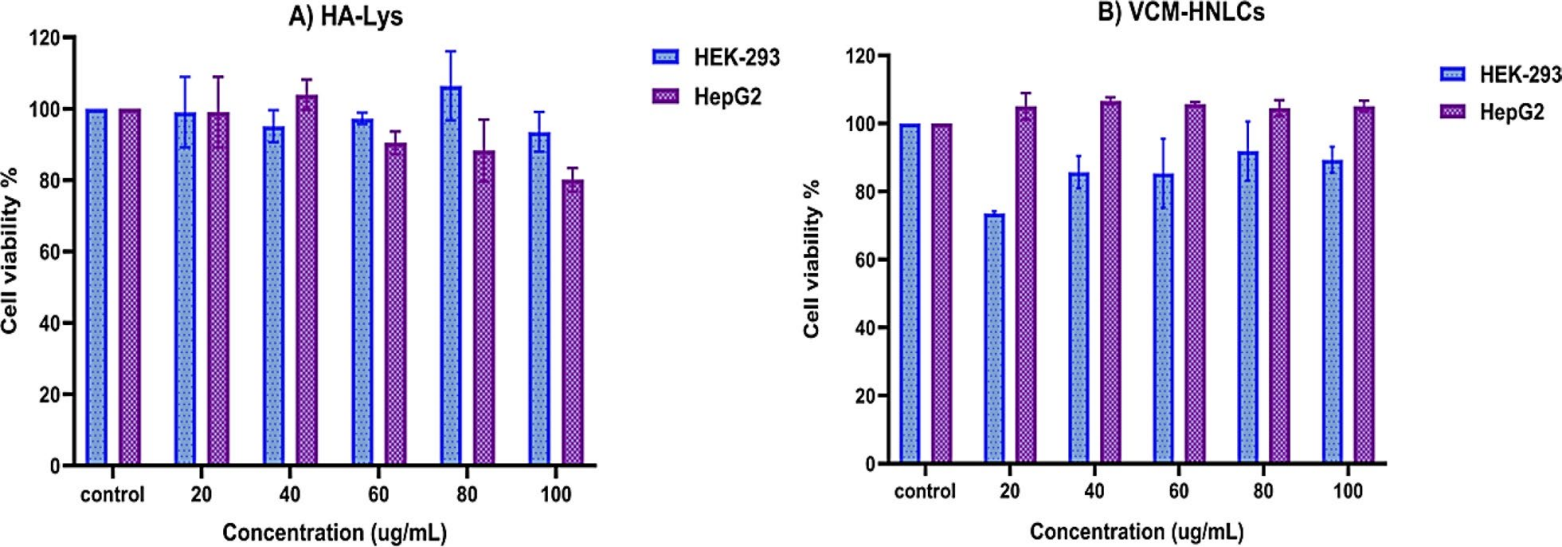
The cell viability % of different concentrations of **A** HA-Lys conjugate and **B** VCM-HNLCs, and controls on HEK293 and HepG2 cell lines. Data are presented as mean ± SD (n = 3)

The biological safety of novel biomaterials and nanocarriers is essential for their application in drug delivery, biomedical, and clinical settings [72]. Therefore, we assessed the cytocompatibility of the synthesized HA-Lys conjugate and its nanoformulation (VCM-HNLCs) across various concentration levels (20–100 μg/mL) using the MTT assay on HEK-293 and HepG2 cell lines as previously described [54]. As depicted in Fig. 3, all tested concentrations maintained cell viability of > 70% across both cell lines. HEK-293 cells exhibited viability ranging from 93.52% to 106.41% for HA-Lys and 73.61% to 91.92% for VCM-HNLCs, while HepG2 cells showed viability ranging from 80.11% to 103.88% for HA-Lys and 104.48% to 106.65% for VCM-HNLCs. Additionally, none of the tested cell lines demonstrated concentration-dependent cytotoxicity within the examined range for HA-Lys and VCM-HNLCs. However, compared to the control group, both the HA-Lys conjugate and VCM-HNLCs formulations showed slightly increased cell viability.

#### Hemolysis

**Fig. 4:**
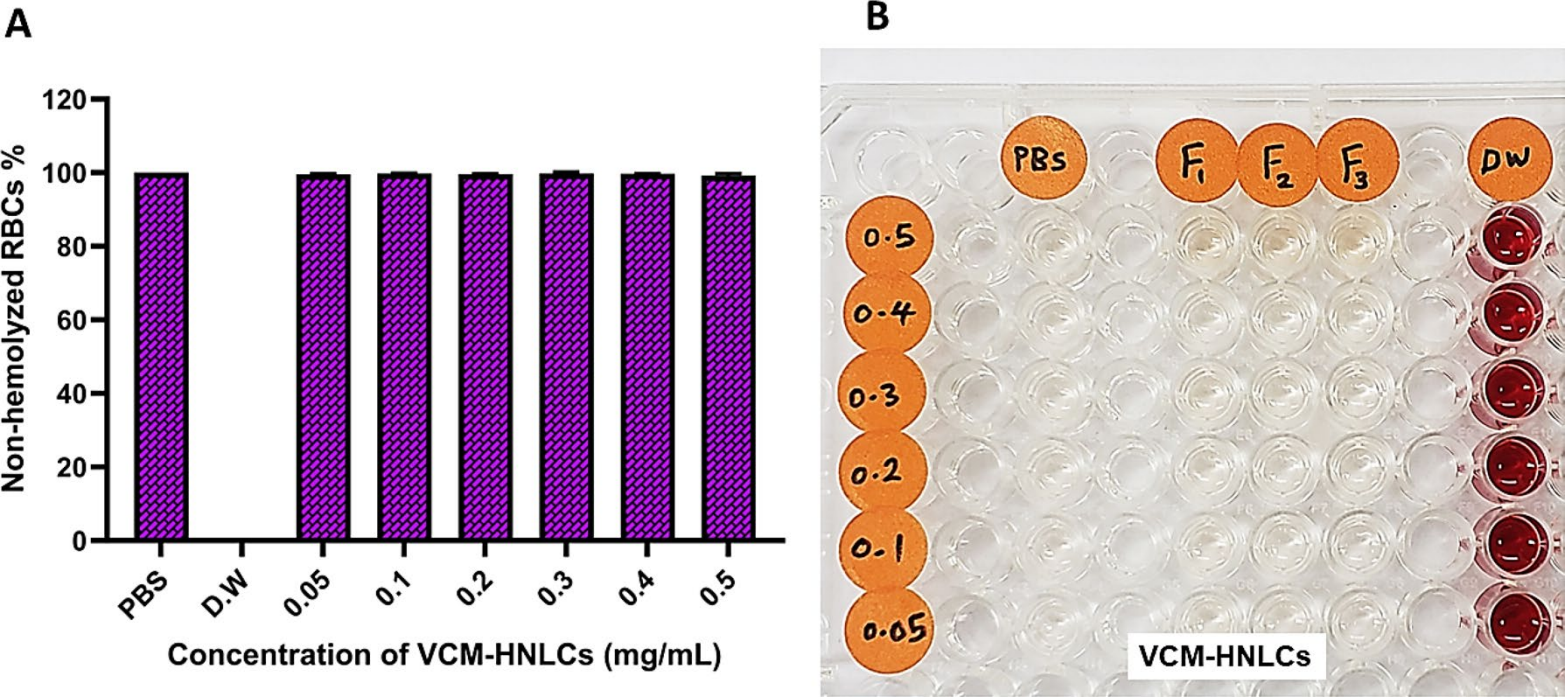
**A**, **B** Non-hemolytic activity of different concentrations of VCM-HNLCs (*DW = distilled water, PBS = phosphate buffer saline pH 7.4). Data are presented as mean ± SD (n = 3)

The hemocompatibility of nanoformulations intended for intravenous administration is essential to ensure their biosafety [73]. Therefore, we assessed the impact of the prepared VCM-HNLCs on erythrocytes using freshly collected and washed sheep blood, and the results are presented in Fig. 4. All concentrations of the VCM-HNLCs exhibited less than 1% hemolysis (refer to Table S3), indicating minimal erythrocyte disruption and a favorable biosafety profile for the nanosystem.

### In vitro binding affinity study

**Fig. 5:**
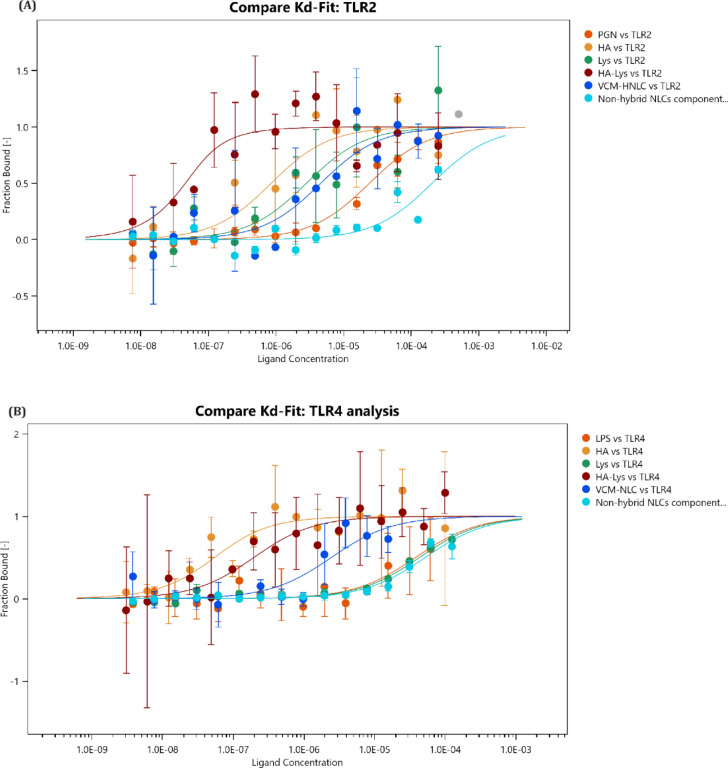
A Comparison view for different ligands fraction bound to **A** TLR2 and **B** TLR4. PGN: Peptidoglycan, LPS: lipopolysaccharide, HA: hyaluronic acid, HA-Lys: hyaluronic acid-lysine conjugate and VCM-HNLCs: vancomycin-loaded hybrid nanostructured lipid carriers. Results represented in mean ± SD (n = 3)

TLRs play a crucial role in immune response initiation by recognizing bacterial endotoxins, a process implicated in both acute and chronic inflammatory conditions like sepsis [74]. Modulating TLR signaling pathways and preventing their interaction with bacterial substrates could mitigate downstream signaling activation, presenting a potential strategy for managing bacterial sepsis. This study employed MST binding affinity analysis to assess the binding affinities of HA, HA-Lys, and VCM-HNLCs to human TLR2 and TLR4 compared to their natural substrates PGN and LPS, respectively. Binding affinities were quantified using dissociation constant (Kd) values (Table 2), and ligand-bound TLR fractions were depicted in Fig. 5.

**Table 2:** The dissociation constant values (K_d_) obtained from the MST analysis for PGN, LPS, individual excipients (HA, Lys and HA-Lys conjugate), VCM-HNLCs and non-hybrid NLCs components binding to TLR2 and TLR4, respectively

Test ligand	Kd (µM)	
	TLR2	TLR4
PGN	23.945 ± 0.880	–
LPS	–	38.145 ± 1.440
HA	0.8237 ± 0.414	0.045 ± 0.750
Lys	2.785 ± 0.740	43.530 ± 2.352
HA-Lys	0.0207 ± 0.848	0.187 ± 0.733
VCM-HNLCs	4.551 ± 0.485	2.443 ± 2.168
Non-hybrid NLCs components	202.1 ± 1.360	52.289 ± 2.206

Data are presented as mean ± SD (n = 3)

Table 2 and Fig. 5A reveal that HA, Lys, and HA-Lys each demonstrated considerable binding affinity to TLR2, with Kd values of 0.8237 ± 0.414, 2.785 ± 0.740, and 0.0207 ± 0.848 µM, respectively, compared to the natural substrate PGN, which had a Kd value of 23.945 ± 0.880 µM (P*-*value < 0.0001). VCM-HNLCs exhibited significantly higher binding affinity to TLR2 than PGN, with a Kd value of 4.551 ± 0.485 µM (P*-*value < 0.0001). In contrast, the non-hybrid NLC components showed markedly lower binding affinity, with more than 40-fold and eightfold higher Kd values compared to VCM-HNLCs and PGN, respectively.

In contrast, the analysis of binding to TLR4 (as shown in Table 2 and Fig. 5B) mirrored the observations for TLR2. Compared to the natural substrate LPS, which had a Kd value of 38.145 ± 1.440 µM, both HA and HA-Lys conjugate exhibited significantly higher binding affinity to TLR4 (P*-*value < 0.0001), with Kd values of 0.045 ± 0.750 and 0.187 ± 0.733 µM, respectively. In contrast, Lys and non-hybrid NLCs components showed the lowest binding affinities, with Kd values of 43.530 ± 2.352 and 52.289 ± 2.206 µM, respectively. Therefore, the strong TLR4 binding of the HA-Lys conjugate is primarily attributed to the HA component of the synthesized polymer. Moreover, VCM-HNLCs demonstrated significantly better binding properties to TLR4 than LPS, with a Kd value of 2.443 ± 2.168 µM (P*-*value < 0.0001).

### In vitro drug release and kinetics

**Fig. 6:**
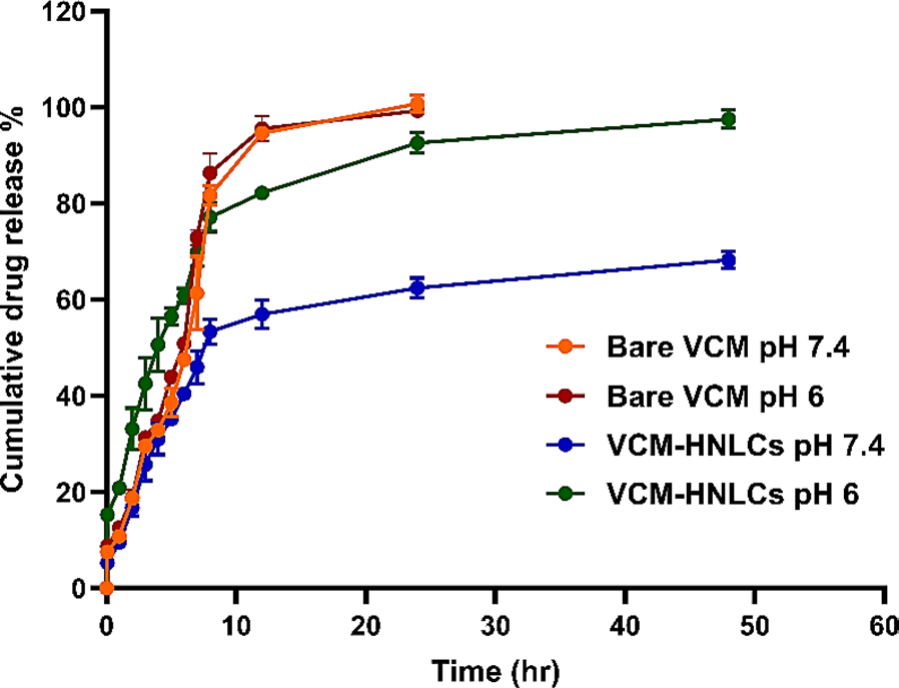
Drug release profile of VCM-HNLCs and bare VCM at pH 7.4 and pH 6.0. All data is represented as mean ± SD (n = 3)

The in vitro release profiles of bare VCM and VCM-HNLCs were evaluated using the dialysis bag method at pH 7.4 and pH 6.0. Figure 6 illustrates that bare VCM exhibited rapid release at pH 7.4, reaching nearly complete release within the first 24 h (100.71%). In contrast, the VCM-HNLCs formulation demonstrated a slower release rate, with approximately 62.42% of VCM released over the same period. This sustained release profile of VCM-HNLCs contrasts with the rapid release of bare VCM, aligning with prior studies reporting around 50% VCM release from nanosystems within the first 24 h [75].

Furthermore, VCM release from VCM-HNLCs was notably higher at pH 6.0 compared to pH 7.4 (P-value = 0.00014). At pH 6.0, 97.48% of VCM was released from the hybrid nanoparticles within 48 h, whereas only 68.23% was released at pH 7.4 during the same timeframe.

To understand the release mechanism of VCM from the prepared HNLCs, cumulative drug release data were analyzed using various mathematical models with the DDSolver program (Table 3). Despite observing higher VCM release from VCM-HNLCs at pH 6.0 compared to pH 7.4, the kinetic behavior remained consistent under both conditions. The Weibull model provided the best fit for describing VCM release at both pH levels, yielding R^2^ values of 0.9507 and 0.9914 and low RMSE values of 5.4391 and 3.1356 for pH 7.4 and 6.0, respectively. The Weibull exponent parameter (β), which characterizes the transport mechanism of drugs through the lipid-polymer matrix, was determined to be 0.472 (< 0.75). This value indicates a Fickian diffusion mechanism for the release of VCM from the HNLCs. Similarly, the release exponent (n) of the Korsmeyer-Peppas model was 0.360 and 0.324 for pH 7.4 and 6.0, respectively, also suggesting Fickian diffusion as the predominant release mechanism. These findings underscore that VCM release from VCM-HNLCs follows a controlled and predictable diffusion process through the lipid-polymer matrix, which is further influenced by pH-responsive properties.

**Table 3:** Kinetics of drug release data for the VCN-HNLCs at pH 7.4 and pH 6

Model	Equation	R^2^		RMSE		Release exponent (n)		β
		pH 7.4	pH 6	pH 7.4	pH 6	pH 7.4	pH 6	
Zero Order	Q = k·t	− 0.1692	− 0.4824	24.2663	37.0378			
First Order	Q = Q0·e^kt^	0.7468	0.9757	11.2864	4.5383			
Higuchi	Q = k·t^1/2^	0.7936	0.7399	10.1912	15.4195			
Korsmeyer-Peppas	Q = k·t^n^	0.8825	0.9103	8.0393	9.9941	0.360	0.324	
Hixson-Crowell	Q^1/3^ = kt + Q_0_^1/3^	0.6497	0.8000	13.2770	13.3885			
Weibull	Q = 1 exp [-(t)a/b]	0.9507	0.9914	5.4391	3.1356			0.472

R^2^: linear regression coefficient, RMSE: Root means square error

### Short-term physical stability study

**Fig. 7:**
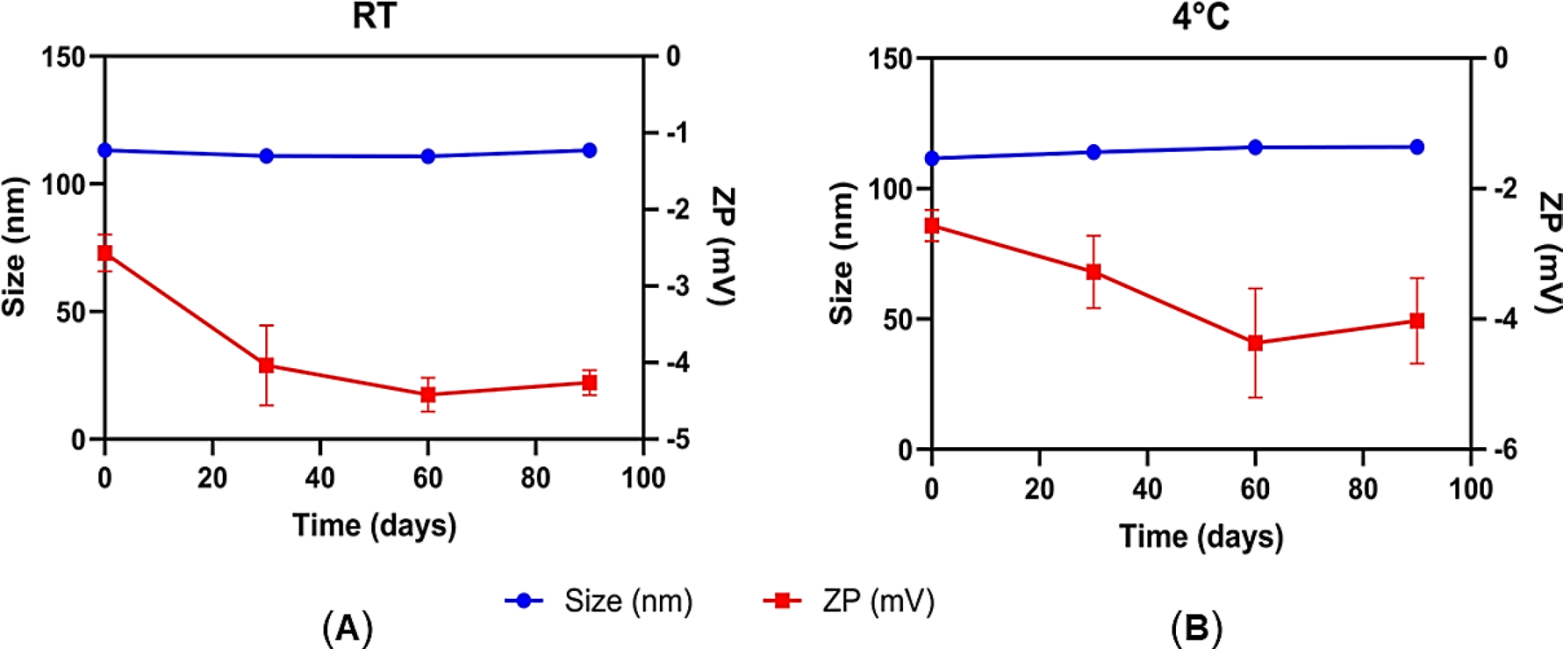
Variations in particle size (nm) and ZP (mV) of VCM-HNLCs during a ninety-day storage at **A** room temperature (RT) and **B** 4 °C. All results are presented as mean ± SD (n = 3)

The size-dependent colloidal stability of the optimized VCM-HNLCs was evaluated by storing the formulation at two different temperatures, 25 °C (room temperature) and 4 °C, for an extended period of 90 days. Figure 7 illustrates the minimal variation in particle size (nm) and ZP (mV) under both conditions, highlighting the consistency of the formulation's physicochemical attributes over time. As summarized in Table S4, the particle size, polydispersity index (PDI), and ZP remained stable, with no statistically significant deviations (P-value > 0.05) throughout the storage period.

### In vitro antibacterial activity studies

#### Determination of minimum inhibitory concentration (MIC)

To investigate the pH-dependent antibacterial activity of VCM-HNLCs against *S. aureus* and MRSA, the minimum inhibitory concentration (MIC) was measured using a broth microdilution method [60]. Table 4 illustrates the MIC values of bare VCM, blank HNLCs, and VCM-HNLCs against *S. aureus* and MRSA at pH 7.4 and pH 6.0. At physiological pH (7.4), VCM-HNLCs demonstrated enhanced antibacterial activity, exhibiting a twofold reduction in MIC values compared to bare VCM against *S. aureus* (1.95 vs 3.9 µg/mL) and MRSA (3.9 vs 7.8 µg/mL) within the first 48 h. Subsequently, bare VCM gradually lost its activity over 72 h, whereas VCM-HNLCs maintained their antibacterial efficacy against both pathogens during the same period. This sustained antibacterial effect of VCM-HNLCs suggests prolonged activity against *S. aureus* and MRSA relative to bare VCM.

**Table 4:** The minimum inhibitory concentration (MIC) values (μg/mL) for *S. aureus* and MRSA at pH 7.4 and pH 6.0, respectively, for bare VCM, blank HNLCs and VCM-HNLCs

S.* aureus* (μg/mL)						
Time (h)	24	48	72	24	48	72
	pH 7.4			pH 6.0		
Bare VCM	3.90	3.90	7.80	3.90	7.80	7.80
Blank HNLCs	16.11	16.11	16.11	16.11	16.11	16.11
VCM-HNLCs	1.95	1.95	1.95	0.98	0.98	0.98

MRSA (μg/mL)						
Time (h)	24	48	72	24	48	72
	pH 7.4			pH 6.0		
Bare VCM	7.80	7.80	15.62	7.80	15.62	15.62
Blank HNLCs	32.22	32.22	32.22	32.22	32.22	32.22
VCM-HNLCs	3.90	3.90	3.90	1.95	1.95	1.95

The test was done in triplicate (n = 3)

VCM: Vancomycin free base; HNLCs: hybrid nanostructured lipid carriers

#### Determination of fractional inhibitory concentration (FIC)

To further assess the combined antibacterial effect of VCM and blank HNLCs in VCM-HNLCs against *S. aureus* and MRSA at pH 7.4 and 6.0, cumulative Fractional Inhibitory Concentration (ΣFIC) values were determined at 24, 48, and 72 h based on MIC values obtained from in vitro antibacterial assays (see Table 5). At pH 7.4, ΣFIC values for *S. aureus* were 0.621, 0.621, and 0.371, indicating additive antibacterial effects for the first 48 h followed by synergistic activity up to 72 h. For MRSA, ΣFIC remained 0.621 across all time points, suggesting additive combination effects. Conversely, at pH 6.0, ΣFIC values were 0.312, 0.187, and 0.187 for *S. aureus*, and 0.311, 0.186, and 0.186 for MRSA, confirming a synergistic effect against both strains at all time intervals.

**Table 5:** ΣFIC values against *S. aureus* and MRSA at pH 7.4 and pH 6.0

FIC against S.* aureus*						
Time (h)	24	48	72	24	48	72
	pH 7.4			pH 6		
FIC (Bare VCM)	0.500	0.500	0.250	0.251	0.126	0.126
FIC (Blank HNLCs)	0.121	0.121	0.121	0.061	0.061	0.061
ΣFIC	0.621	0.621	0.371	0.312	0.187	0.187
Interpretation	Additive	Additive	Synergy	Synergy	Synergy	Synergy

FIC against MRSA						
Time (h)	24	48	72	24	48	72
	pH 7.4			pH 6		
FIC (Bare VCM)	0.500	0.500	0.250	0.250	0.125	0.125
FIC (Blank HNLCs)	0.121	0.212	0.121	0.061	0.061	0.061
ΣFIC	0.621	0.621	0.621	0.311	0.186	0.186
Interpretation	Additive	Additive	Additive	Synergy	Synergy	Synergy

#### Flow cytometry

**Fig. 8:**
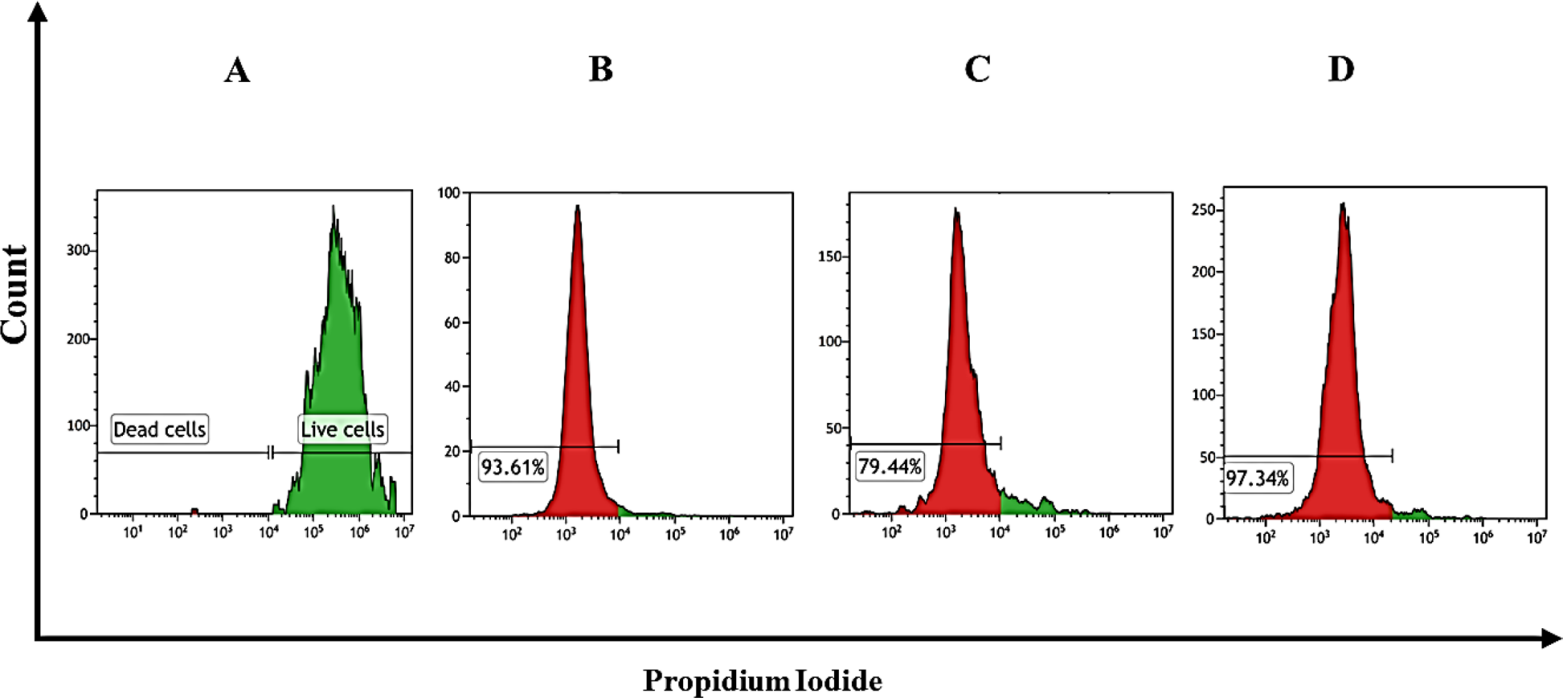
Histogram of cell count versus propidium iodide (PI) uptake. **A** corresponds to the untreated MRSA (live cells); **B**, **C** and **D** indicate the proportion of dead MRSA cells following incubation with bare VCM at its MIC (7.8 μg/mL), bare VCM in NPs MIC (3.9 μg/mL) and VCM-HNLCs at its MIC (3.9 μg/mL), respectively. The experiment was conducted in triplicate (n = 3)

The impact of bare VCM and VCM-HNLCs on MRSA cell viability was assessed using a flow cytometry technique described in previous literature with minor adaptations [61]. Antibiotic activity induces significant changes in bacterial morphology and the cell division cycle, which can be discerned using specific dyes [76]. Propidium iodide (PI), a non-permeant fluorescent dye, was utilized to identify damaged bacterial cells, while Syto9, a green, fluorescent dye permeant to nucleic acids, was used to distinguish live cells within the population. Flow cytometry data were analyzed using Kaluza-2.20 software (Beckman Coulter, USA), where two gates were defined to differentiate viable (green) and dead (red) cells in the sample. Figure 8 illustrates the cell count versus PI uptake histogram for MRSA, indicating that 93.61 ± 0.33% and 97.34 ± 2.71% of MRSA cells were non-viable after exposure to bare VCM (Fig. 8B) and VCM-HNLCs (Fig. 8D), respectively, at their respective MICs (7.8 and 3.9 μg/mL). This demonstrates that VCM-HNLCs achieved superior bactericidal activity compared to bare VCM at a twofold lower inhibitory concentration (P-value = 0.003). Conversely, only 79.44 ± 1.13% of MRSA cells were non-viable (Fig. 8C) when treated with bare VCM at the same concentration as VCM-HNLCs (3.9 μg/mL), significantly lower than that observed with the hybrid NPs (P-value < 0.0001).

#### Determination of in vitro antibiofilm activity

##### Crystal violet assay

**Fig. 9:**
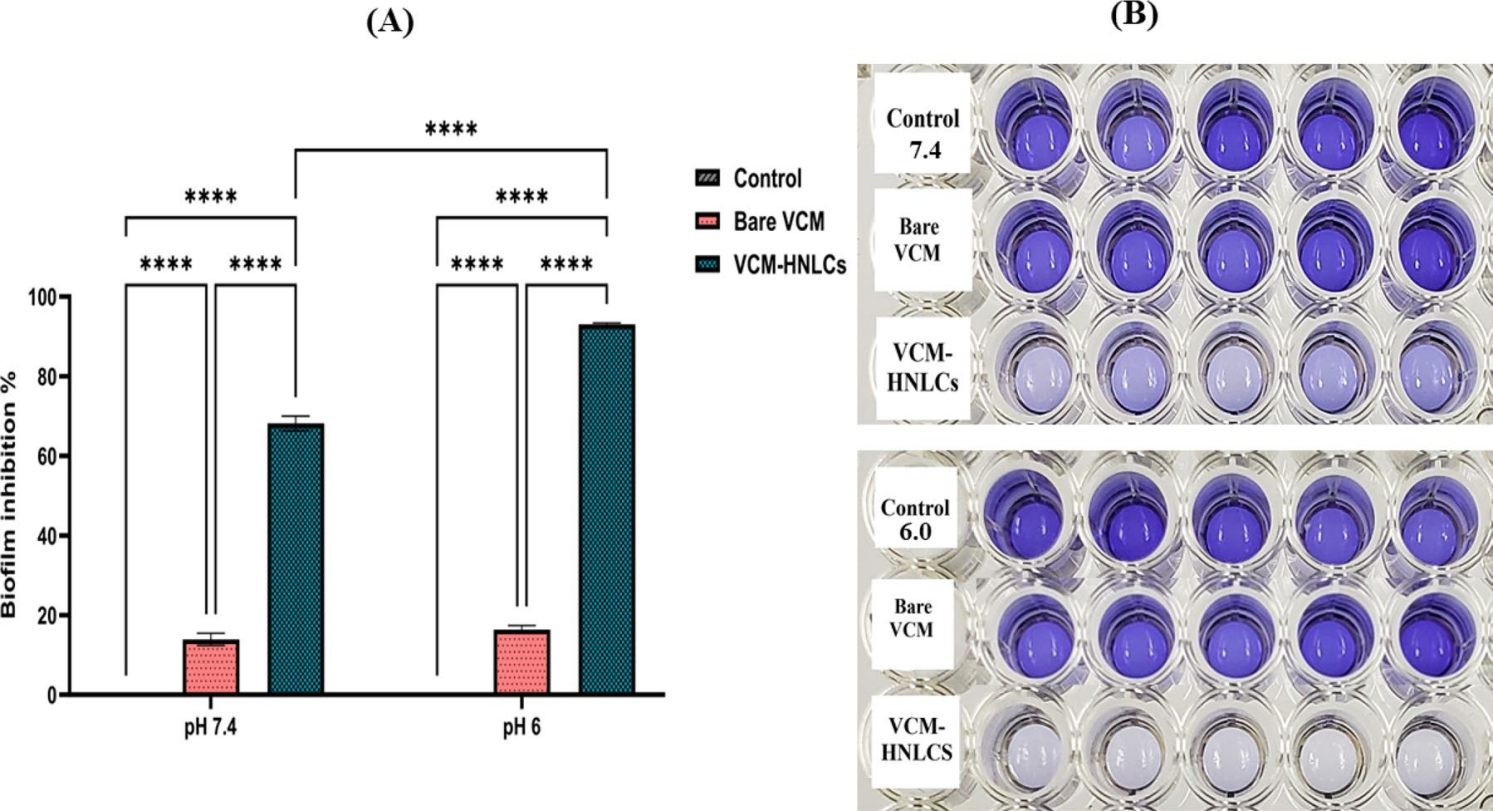
**A** Percentage of biofilm growth suppression following the exposure of MRSA biofilms to bare VCM, VCM-HNLCs and controls (PBS pH 7.4 and 6.0). Each result is shown as mean ± SD (n = 3) (P-values **** < 0.0001). **B** MRSA biofilm growth reduction after exposure to bare VCM and VCM-HNLCs at PBS pH 7.4 and 6.0, as indicated by changes in the crystal violet intensity

The efficacy of the prepared VCM-HNLCs formulation against MRSA biofilms was evaluated using crystal violet staining. Mature MRSA biofilms were treated with PBS at pH 7.4 and 6.0 (as controls), bare VCM solution, and VCM-HNLCs formulation for 24 h. Following treatment, the biofilms were stained with crystal violet, and changes in staining intensity were assessed to quantify biofilm growth inhibition. The results, depicted in Fig. 9, indicate that VCM-HNLCs exhibited superior efficacy in eradicating MRSA biofilms at pH 7.4, reducing biofilm biomass by approximately fivefold (68.17 ± 1.83%) compared to bare VCM, which achieved only 14.25 ± 2.62% biofilm elimination. At pH 6.0, VCM-HNLCs reduced biofilm biomass by approximately sixfold (93.51 ± 0.83%), significantly outperforming bare VCM, which eradicated only 16.41 ± 0.80% of the biofilm. Furthermore, VCM-HNLCs demonstrated notable pH-responsive antibiofilm activity, exhibiting around 1.4-fold higher efficacy against MRSA biofilms at pH 6.0 compared to pH 7.4.

##### Florescence microscope assay

**Fig. 10:**
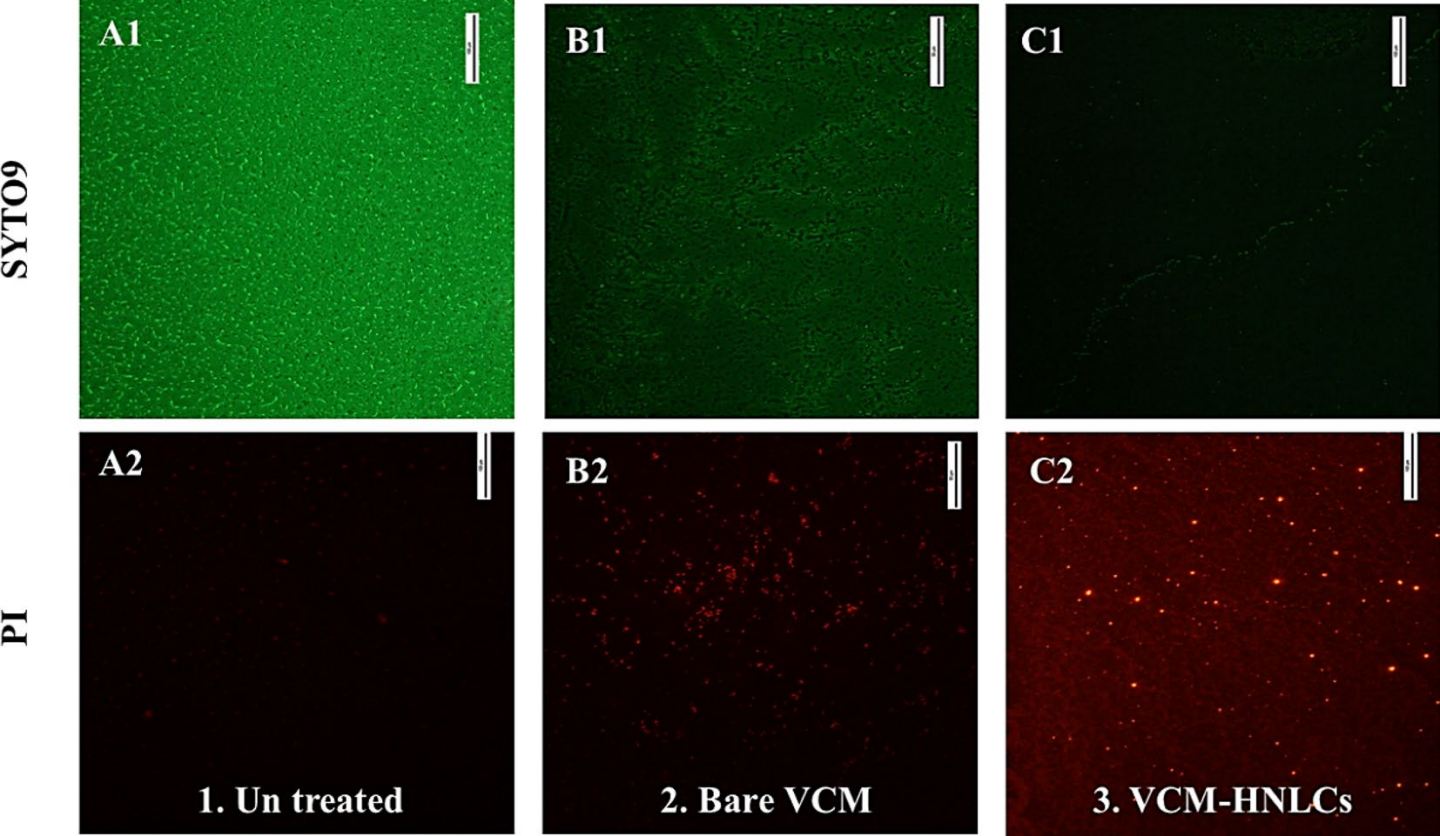
Images of the fluorescence microscope assay of MRSA biofilms stained with Syto9 and PI. **1**. Untreated MRSA biofilms **2.** Bare VCM-treated biofilms, and **3.** VCM-HNLCs treated biofilms, respectively. (Scale bar = 100 µm)

The efficacy of VCM-HNLCs in eradicating mature MRSA biofilms was further validated using fluorescence microscopy. Mature biofilms cultured on coverslips were stained with Syto9 and PI solutions in the dark for 30 min. After washing off the dyes, coverslips were inverted onto glass slides for observation. As shown in Fig. 10, untreated MRSA biofilms showed substantial Syto9 fluorescence across the coverslip (Fig. 10A1), indicating intact bacterial cell membranes [77, 78]. Conversely, there was negligible fluorescence in MRSA biofilms stained with PI (Fig. 10A2), as PI cannot penetrate live cells [79, 80]. Biofilms treated with bare VCM exhibited a minimal reduction in Syto9 fluorescence compared to untreated biofilms (Fig. 10B1), while a slight fluorescence was observed in PI-stained biofilms (Fig. 10B2), suggesting partial permeation of the drug through the biofilm and partial bacterial cell death. In contrast, treatment of MRSA biofilms with VCM-HNLCs significantly reduced Syto9 fluorescence intensity (Fig. 10C1), indicating a decrease in viable cells within the biofilm. Interestingly, despite PI's inability to permeate intact cells, biofilms treated with VCM-HNLCs exhibited intense PI fluorescence (Fig. 10C2), suggesting disruption of MRSA cell membranes and enhanced PI penetration to bind to DNA.

#### Time-killing assay (TKA)

**Fig. 11:**
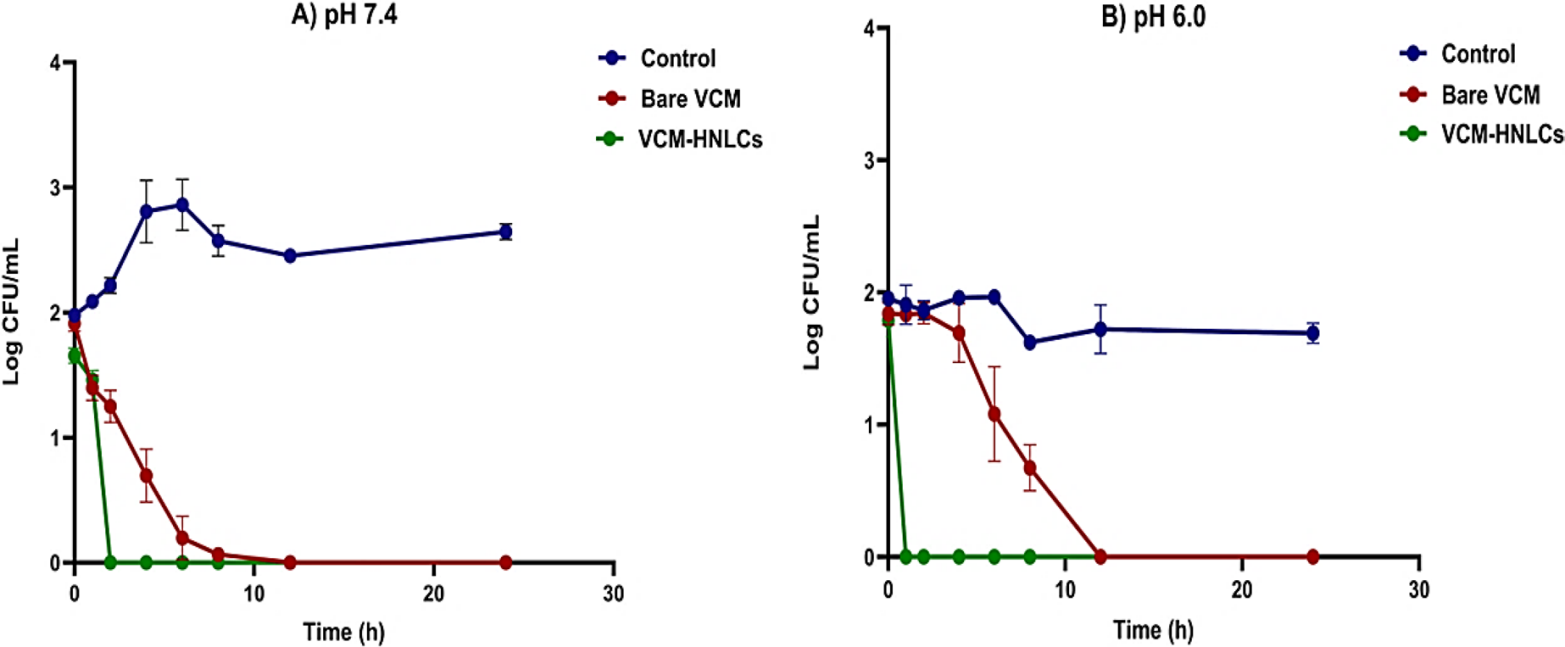
MRSA time-killing kinetics when exposed to bare VCM, VCM-HNLCs and **A** PBS pH 7.4 and **B** pH 6.0 (as controls). The findings are shown as mean ± SD (n = 3). At pH 6, VCM-HNLCs demonstrated more enhancement of VCM-killing kinetics (P-value < 0.0001)

The time-killing assay was conducted to assess the rate of bacterial eradication following 24-h exposure of MRSA to bare VCM and VCM-HNLCs formulation solution (5 × MIC) at pH 7.4 and 6.0 (Fig. 11). Both treatments significantly reduced the number of MRSA colonies under both pH conditions. Notably, VCM-HNLCs demonstrated superior efficacy compared to bare VCM, achieving complete eradication of MRSA bacteria within the first 2 h at pH 7.4 and within 1 h at pH 6.0. In contrast, bare VCM exhibited slower bactericidal kinetics over the 24-h period at both pH levels. Hence, VCM-HNLCs showed a significantly faster bactericidal effect against MRSA at a twofold and fourfold lower concentration than bare VCM at pH 7.4 and 6.0, respectively (P-value < 0.0001).

#### Efflux pump inhibition assay

**Fig. 12:**
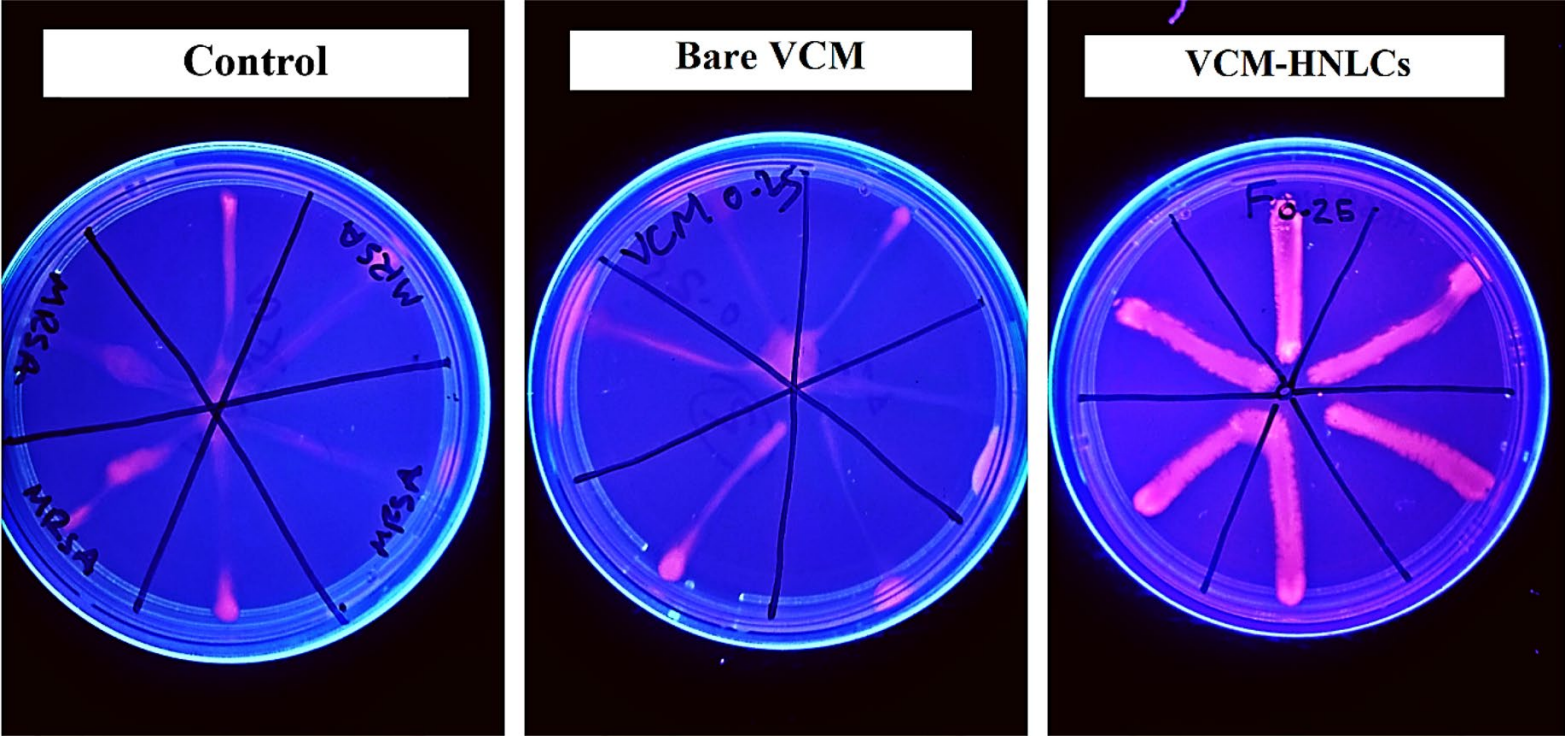
Efflux pump inhibition effect of VCM-HNLCs against MRSA compared to the bare VCM at 0.25 × MIC

MRSA has been observed to develop resistance to antibiotics through efflux-mediated mechanisms, which involve genetic mutations that reduce bacterial susceptibility to antimicrobial agents [81]. Consequently, this study investigated the ability of subinhibitory concentrations of VCM-HNLCs (0.25 × MIC) to inhibit the overexpression of efflux pumps induced by MRSA, using ethidium bromide (EtBr) efflux pump cartwheel method [66]. EtBr binds to intracellular DNA and fluoresces within bacterial cells, thus indicating efflux pump activity. As depicted in Fig. 12, MRSA treated with VCM-HNLCs displayed increased fluorescence intensity, suggesting substantial accumulation of EtBr within bacterial cells, compared to those treated solely with bare VCM.

### In vitro antioxidant study

Excessive production of ROS during inflammation contributes significantly to oxidative stress, a major factor in the increased mortality associated with sepsis [82]. To evaluate the antioxidant properties of various concentrations of individual excipients (TS, Lys, and HA-Lys) and VCM-HNLCs, a DPPH radical scavenging assay was employed [67].

**Fig. 13:**
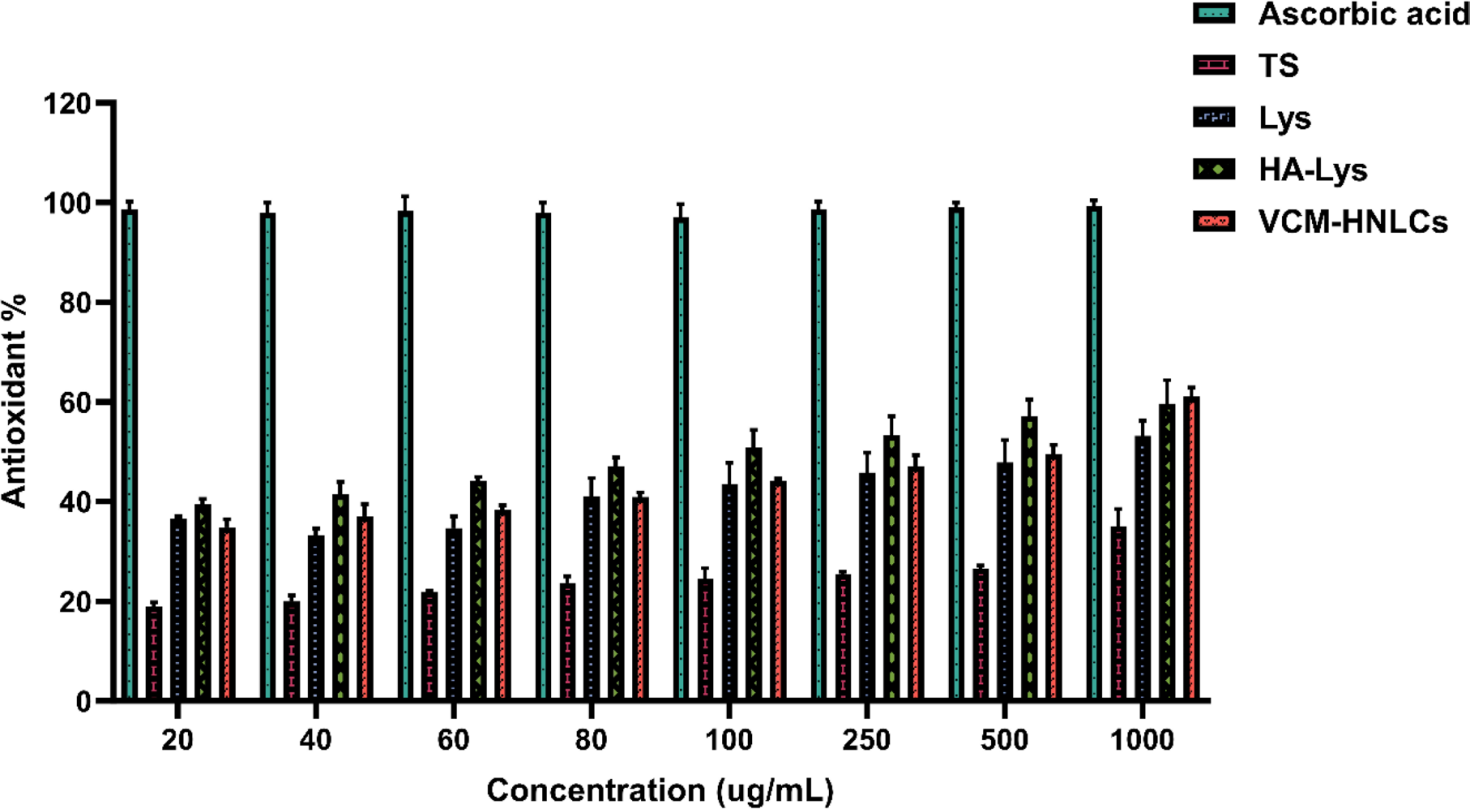
The antioxidant activity (%) of various concentrations (µg/mL) of TS, Lys, HA-Lys conjugate and VCM-HNLCs according to a DPPH radical scavenging assay in comparison to ascorbic acid (positive control). Data in the graph are presented as mean ± SD (n = 3). (TS: Tocopherol succinate, Lys: L-Lysine, HA-Lys: hyaluronic acid-lysine conjugate, VCM-HNLCs: vancomycin-loaded hybrid nanostructured lipid carriers, DPPH: 2.2-diphenyl-1-picrylhydrazyl)

Figure 13 presents the antioxidant activity percentages of ascorbic acid (positive control), TS, Lys, HA-Lys conjugate, and VCM-HNLCs after incubation with DPPH solution at 37°C in the dark. Specifically, the scavenging activities were measured as 34.98 ± 3.59%, 53.21 ± 2.30%, 59.69 ± 4.68%, and 61.08 ± 1.87% for TS, Lys, HA-Lys conjugate, and VCM-HNLCs, respectively. Among the individual excipients, Lys and HA-Lys conjugate demonstrated higher scavenging activity compared to TS across all concentrations tested.

#### In vitro anti-inflammatory studies

In order to address the dual challenge of eliminating causative bacteria and modulating the immune response mediated by bacterial toxins like LPS in bacterial sepsis treatment, we assessed the anti-inflammatory activity of VCM-HNLCs formulations. Using the HepG2 cell line [55, 68], we examined the anti-inflammatory effects of both HA-Lys conjugate and VCM-HNLCs formulations against LPS-induced inflammation. This evaluation involved measuring cell viability through the MTT assay and quantifying IL-6 levels using enzyme-linked immunosorbent assay (ELISA).

#### Cytoprotective effect against LPS-induced cytotoxicity

**Fig. 14:**
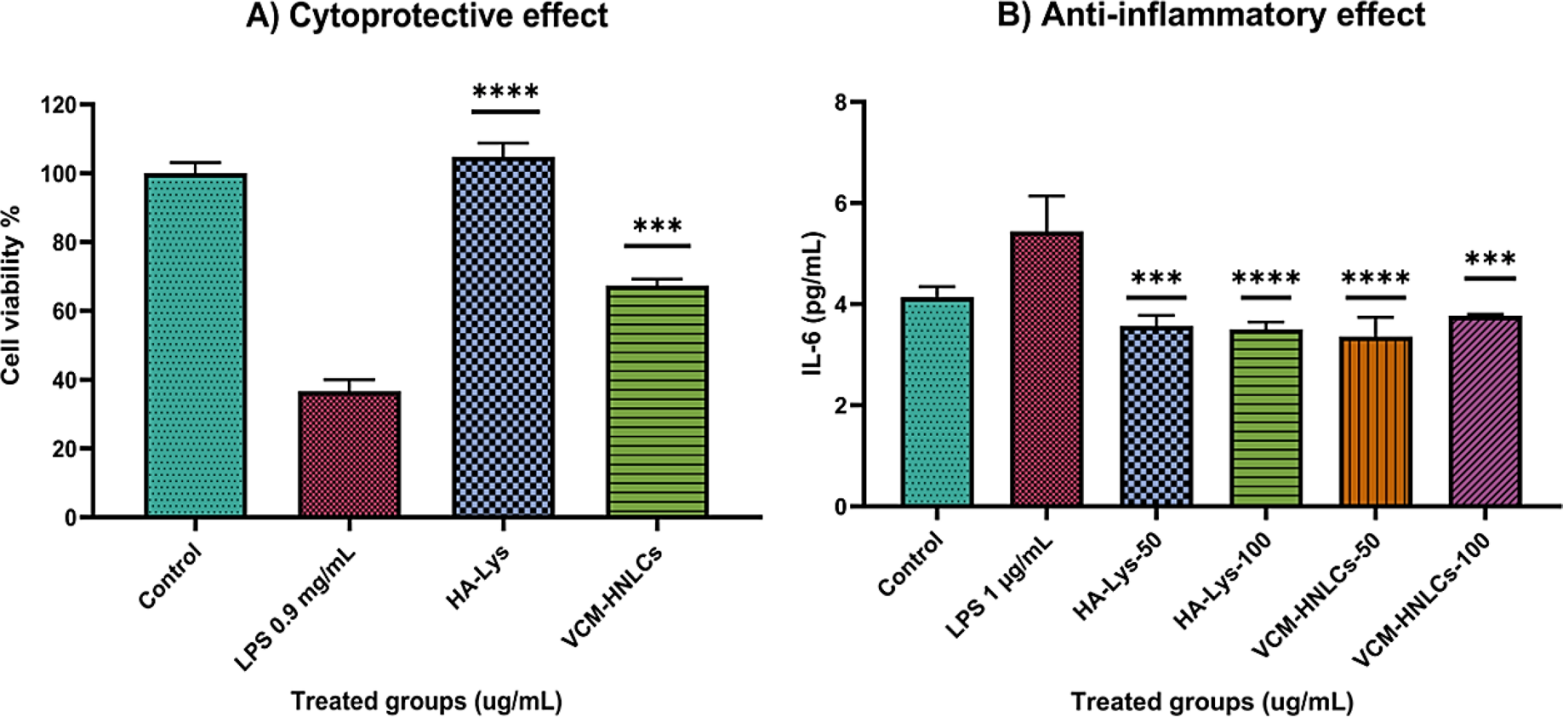
**A** The Cytotoxicity of LPS on HepG2 cells and the protective effects of HA-Lys and VCM-HNLCs as measured by MTT assay, and **B** The effect of HA-Lys and VCM-HNLCs on IL-6 expression induced by LPS. (****P-value < 0.0001, ***P*-*value = 0.0002, in comparison with the LPS-treated group). Data in the graph are presented as mean ± SD (n = 3)

As shown in Fig. 14A, exposure to LPS (0.9 mg/mL) resulted in a significant reduction in HepG2 cell viability to 36.70 ± 3.30% compared to the control group (P-value < 0.0001). However, supplementation with HA-Lys or VCM-HNLCs formulations (100 µg/mL) notably improved cell viability to 100.84 ± 3.91% and 67.34 ± 1.87%, respectively, relative to the control. Importantly, treatment with HA-Lys and VCM-HNLCs formulations maintained cell viability compromised by LPS by 64.14% (P-value < 0.0001) and 30.64% (P-value = 0.0002), respectively, compared to the LPS-treated group. These findings underscore the potential of HA-Lys and VCM-HNLCs formulations to mitigate LPS-induced cytotoxicity and suggest their efficacy in reducing inflammation-associated cell damage.

#### Anti-inflammatory activity against LPS-induced inflammatory responses (measurement of IL-6 levels)

To further validate the anti-inflammatory efficacy of synthesized HA-Lys and VCM-HNLCs formulations, their impact was evaluated on the expression of the pro-inflammatory cytokine IL-6 in LPS-stimulated HepG2 cells. As depicted in Fig. 14B, treatment with 1 µg/mL of LPS significantly increased IL-6 levels. However, treatment with varying concentrations of HA-Lys or VCM-HNLCs formulations (50 and 100 µg/mL) markedly reduced IL-6 levels compared to the group treated solely with LPS (P-value < 0.0001).

### In vivo studies

**Fig. 15:**
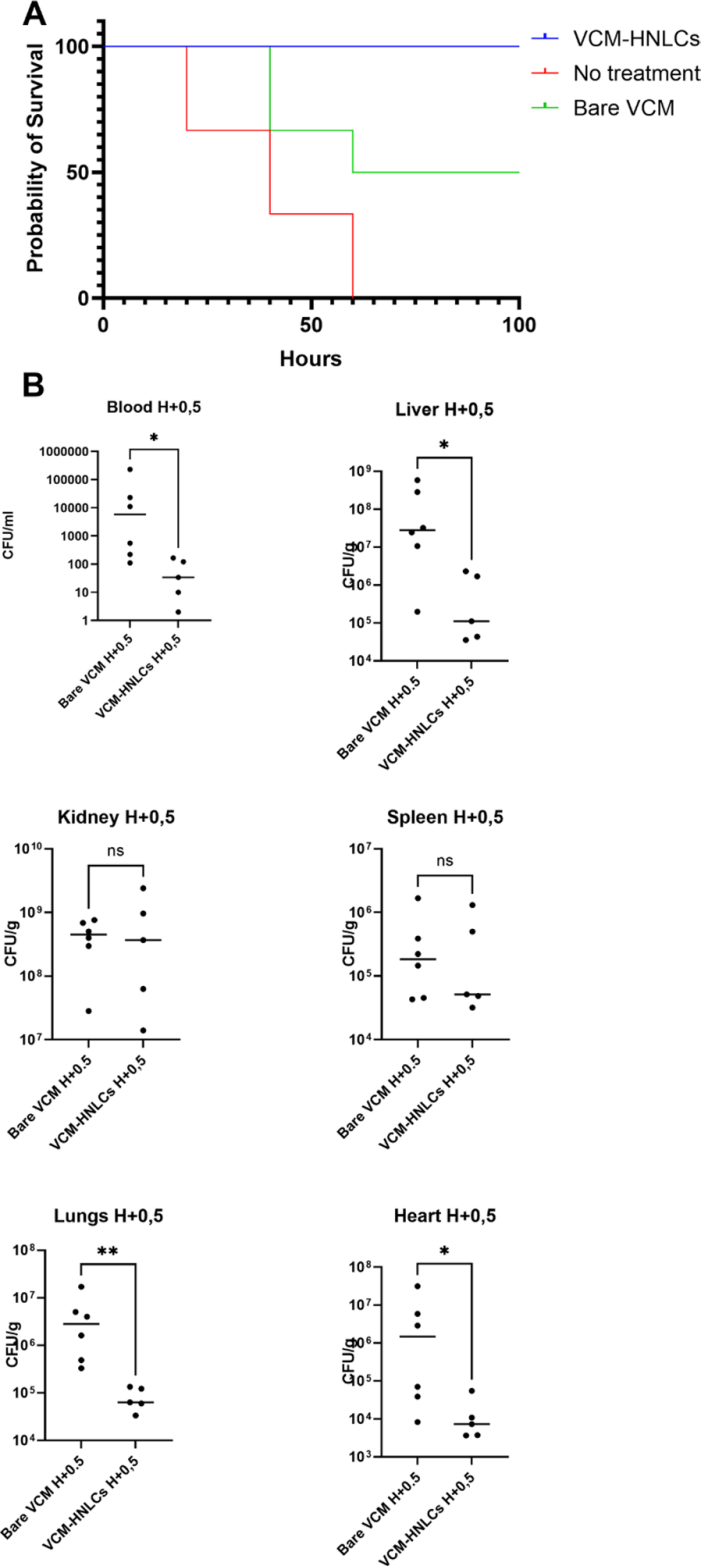
Survival and CFUs recovered in different organs in MRSA-induced systemic bacteremia. **A** Kaplan–Meier survival curves depicting the survival of different treatment groups following MRSA infection. **B** Comparison of CFUs recovered in blood, liver, kidney, heart, lungs, and spleen after treatment with bare VCM and VCM-HNLCs.

Given that VCM-HNLCs have demonstrated enhanced in vitro antibacterial activity and excellent biocompatibility, we proceeded to assess the protective effects of both free VCM and VCM-HNLCs on MRSA-induced sepsis in mice by evaluating the survival rate at 100 h post-infection. As depicted in Fig. 15A, the MRSA-infected, unencapsulated VCM group and VCM-HNLCs exhibited a 0% survival rate by 60 h post-infection, 50% survival rate after 100 h after infection and 100% survival of the infected mice at 100 h post-infection respectively, as illustrated by the Kaplan–Meier curve in Fig. 15A.

Following survival analysis, surviving animals were euthanized, and CFUs from various organs were isolated to evaluate bacterial burden. As revealed in Fig. 15A, in comparison to free VCM, treatment with VCM-HNLCs led to a significant reduction in MRSA burden across multiple organs: blood (798-fold reduction, P-value = 0.0173), liver (217-fold reduction, P-value = 0.0173), lungs (59-fold reduction, P-value = 0.0043), and heart (500-fold reduction, P-value = 0.0303). However, there was no significant reduction in kidney (0.57-fold, P-value > 0.9999) and spleen (1.27-fold, P-value = 0.9307), as shown in Fig. 15B.

## Discussion

The current therapy for mitigating bacterial-induced sepsis is inadequate in achieving satisfactory clinical outcomes. One strategy to enhance therapeutic efficiency against sepsis involves improving drug delivery systems through the use of multifunctional biomaterials. In this study, a Hyaluronic acid-lysine (HA-Lys) conjugate was synthesized, thoroughly characterized, and subsequently employed in the development of multifunctional pH-responsive and biomimetic hybrid nanostructured lipid carriers (HNLCs) designed to target bacterial sepsis. The HA-Lys conjugate was successfully synthesized using a one-step amidation reaction and characterized using FTIR and ^1^H NMR spectroscopy.

Following the successful synthesis of the HA-Lys conjugate, the biomaterial was employed in the formulation of a hybrid nanosystem utilizing the hot homogenization-ultrasonication technique. The initial formulation optimization involved the screening process for the suitable surfactant (Table S2), followed by optimizing other formulation parameters. As shown in Table 1, increasing the lipid-to-surfactant mass ratio from 1:0.5 to 1:2 resulted in a decrease in the average particle diameter, EE %, and DL % w/w with a slight increase in PDI. This can be attributed to the surfactant molecules available to cover and stabilize the lipid-water interfaces formed during homogenization, thus decreasing interfacial tension and yielding NPs with smaller particle sizes [83]. These findings align with previous research studies that have also observed a decrease in the particle size of NLCs at higher surfactant concentrations [84–86]. Conversely, the reduction in EE % and DL % w/w of the prepared VCM-HNLCs with increasing surfactant content may be attributed to the high hydrophilicity of the loaded VCM, causing the drug to partition out of the lipid system into the surrounding aqueous medium [87]. On the other hand, increasing the drug: lipid mass ratio from 1:3 to 1:5 resulted in an increase in EE % and DL % w/w without significant effects on the size, PDI and ZP. This enhanced EE % could be due to the increase in lipid content, which improves the dispersion of VCM within the lipid matrix while minimizing its leakage from the NPs [20, 88]. Moreover, increasing the lipid: polymer mass ratio from 1:0.05 to 1:0.15 resulted in an increase in the EE % without any significant effects on the other parameters among all the samples tested. These findings were consistent with previously published studies, where increasing the lipid: polymer mass ratio led to an increase in particle size and size distribution of the NPs [89, 90].

Despite the fact that all formulations revealed relatively low ZP values, it is worth noting that ZP alone does not provide a complete understanding of the phenomena involved in particle stability. According to the DLVO theory, colloid stability relies on the balance between van der Waals attractive forces and electrostatic repulsive forces resulting from the electric double layer. While ZP indicates the strength of electrostatic repulsion, it does not provide insight into the contribution of attractive van der Waals forces [91]. Therefore, a comprehensive evaluation of colloidal stability must consider not only ZP but also other factors, such as particle size distribution and surface charge. Furthermore, the short-term stability study demonstrated that the nanocarriers retained their structural integrity and functional properties under typical storage conditions, highlighting their potential as reliable drug delivery systems. This stability is crucial for ensuring the reproducibility and efficacy of the formulation in pharmaceutical applications. Overall, these findings suggest that VCM-HNLCs are promising candidates for safe and effective drug delivery with stable storage conditions.

The pH responsiveness of the VCM-HNLCs was validated by charge switching from negative to positive charge (Fig. 1). This charge shift is likely attributed to the protonation of the primary amino group of OLA, resulting in a positive ZP at low pH values. It is anticipated that this change in surface charge from negative to positive may promote drug release and enhance adherence of VCM-HNLCs to the negatively charged bacterial cell wall, thereby increasing antibacterial efficacy [92, 93]. This result was further confirmed by evaluating the drug release profile of the system at pH 7.4 and 6.0. The result indicated a sustained release profile for the VCM-HNLCs formulation at both pHs in contrast to the bare VCM. The delayed release behavior of VCM from the VCM-HNLCs may be caused by the diffusion of the entrapped VCM molecules through the lipid-polymer matrix, which retains the antibiotic for a longer period of time [89]. The system demonstrated pH-dependent drug release behavior, with significantly higher VCM release from VCM-HNLCs at pH 6.0 compared to pH 7.4 (P-value = 0.00014). This enhanced drug release at acidic pH is due to the confirmational changes and destabilization of the NPs as a result of OLA protonation at lower pH values, which accelerates drug release profiles [94]. These findings were in accordance with previously reported studies, showing that VCM released from pH-responsive nanosystems was greater and occurred more quickly at pH 6.0 than it was at physiological pH 7.4. This pH-triggered VCM release behavior is essential for better drug protection at the physiological pH and improved localization at the infection sites (acidic pH), which helps in enhancing the antibacterial activity [95, 96].

The biosafety of the VCM-HNLCs was determined via MTT assay and hemolysis assay. None of the tested cell lines exhibited concentration-dependent cytotoxicity across the concentration range examined for HA-Lys and VCM-HNLCs. However, in contrast to the control group, the HA-Lys conjugate and VCM-HNLCs formulations demonstrated marginally increased cell viability. This could be attributed to the proliferative action of HA [97], as well as long-chain fatty acid derivatives such as OLA [98]. These findings were consistent with the International Standard Organization (ISO) limit for biosafety of biomedical materials (≥ 70% cell viability) [99] and support the cytocompatibility and potential use of the prepared VCM-HNLCs for nano antibiotic delivery. Additionally, the non-hemolytic nature of VCM-HNLCs, as shown in Fig. 4, confirms the suitability of the nanocarrier for parenteral administration.

Activation of TLRs by bacterial endotoxins is a critical step in triggering immune response, which also contributes to the development of chronic and acute inflammatory conditions like sepsis [74]. Therefore, altering TLR signaling pathways and blocking their binding to their natural bacterial substrates may help reduce the subsequent activation of downstream signaling molecules and could be a potential strategy for treating bacterial sepsis. In this research, an MST binding affinity analysis was conducted to evaluate the binding affinity of HA (a known ligand of TLR2 and TLR4), as well as Lys, HA-Lys, and VCM-HNLCs to human TLR2 and TLR4 compared to their natural substrates PGN and LPS, respectively. The obtained results confirmed significantly enhanced binding affinity of the nanocarrier to TLR2 and TLR4 compared to their natural substrates. The inclusion of HA-Lys in the VCM-HNLCs formulation may be responsible for the binding affinity and consequent TLR2 and TLR4 sensitivity. The change in binding affinity of VCM-HNLCs compared to HA-Lys might be attributed to the conformational shift in the HNLCs structure resulting from the self-assembly of the nanosystem. Accordingly, the findings of the binding study demonstrate a direct and robust binding of HA-Lys and VCM-HNLCs to both human TLR2 and TLR4, which could potentially inhibit the binding of bacterial toxins to their corresponding receptors and suppress the inflammatory response triggered by TLRs. Therefore, HA-Lys and its nanoformulation (VCM-HNLCs) may be suggested as an adjuvant and potential therapeutic nanocarrier for treating sepsis resulting from mixed bacterial infections.

After evaluating drug release, the pH-dependent antimicrobial activity of the system was determined against *S. aureus* and MRSA by measuring the minimum inhibitory concentration (MIC). The hybrid NPs also exhibited superior and sustained antibacterial activity compared to bare VCM, with VCM-HNLCs revealing significantly lower MIC at pH 6.0 compared to pH 7.4 against both tested pathogens. This effect could be attributed to the combined and inherent antibacterial properties of Tween 80 [100], OLA [101] and TS [46] against gram-positive bacteria. The enhanced and sustained antibacterial activity of VCM-HNLCs over the bare VCM under normal conditions may be due to numerous factors including (a) the gradual and extended-release profile of VCM from the hybrid NPs resulting in long-lasting antibacterial efficacy [102], (b) the smaller particle size of the NPs which facilitates better adsorption and effective cell wall penetration [103], and (c) the potential effect as a result of the intrinsic antibacterial activity of the nanoformulation components. Furthermore, the improved antimicrobial activity of the prepared VCM-HNLCs at the acidified infection pH may be due to the rapid VCM release from the HNLCs at pH 6.0 and the surface charge switch from negative to positive charge due to the protonation of OLA, which improves the adhesion of VCM-HNLCs to the negatively charged bacterial cell wall leading to superior antibacterial efficacy. The enhanced antibacterial activity associated with the antibiotic-loaded hybrid nanocarrier may help reduce the effective treatment dose without compromising therapeutic outcomes [104]. Moreover, the frequency of VCM administration could be decreased, which reduces the dose-related toxicity and improves patient compliance [105]. A comparable pattern of improved and sustained pH-responsive antibacterial activity has been documented previously for VCM-loaded NLCs [20] and VCM-loaded SLNs [58] against both *S. aureus* and MRSA. This synergistic effect suggests that a lower dosage of VCM-HNLCs may suffice to achieve equivalent therapeutic efficacy compared to free VCM, potentially enhancing antibacterial effectiveness while minimizing associated toxicity and side effects.

Both the bacterial live/dead cell assay and time-kill assay have demonstrated that VCM-HNLCs outperform bare VCM in reducing MRSA burden at significantly lower concentrations and at a faster rate. These findings strongly indicate the potential enhancement of antibacterial effectiveness against MRSA with VCM-HNLCs, potentially enabling the reduction of VCM dosage in treating MRSA infections without compromising therapeutic efficacy [106]. Moreover, conventional VCM therapy is often characterized by slow and time-dependent antibacterial efficacy, inadequate penetration into solid organs, and suboptimal pharmacokinetic properties, contributing to high rates of antimicrobial resistance, treatment failure, and toxicity [107]. Therefore, these findings underscore the potential of VCM-HNLCs as effective nanocarriers to improve the bacterial killing kinetics of antibiotics, achieve sustained release in the body, and potentially enable successful therapy with lower dose regimens against MRSA infections and associated sepsis.

Biofilm formation is one of the survival strategies of many microorganisms, including bacteria. They play a significant role in the pathogenesis of various bacterial infections, particularly MRSA infections, rendering them more resistant to the host's immune system and antimicrobial treatments [108]. Therefore, developing a nanoantibiotic delivery system with improved antibacterial activity and the ability to successfully remove biofilms to combat MDR bacterial infections and sepsis would be beneficial. The efficiency of the prepared VCM-HNLCs formulation against MRSA biofilms was explored using both the crystal violet staining assay and fluorescence microscopy. Both approaches elucidated the superiority of the prepared HNLCs in eliminating MRSA biofilms compared to bare VCM. This improved antibiofilm activity could be due to the combined effects of TS and Tween 80, as well as the pH-responsive property of the nanosystem. It has been reported that TS has vital and intrinsic anti-adhesion properties against *S. aureus* biofilms. Therefore, it could act synergistically with VCM to affect pathogen adhesion and biofilm formation, making it a suitable pharmaceutical adjuvant for antibiotic therapy [45, 46, 109]. Furthermore, Tween 80 has been reported to possess potential antibiofilm action against various pathogenic bacteria, including MRSA. It can reduce bacterial adhesion to surfaces and facilitate biofilm elimination by disturbing the biofilm matrix and increasing its permeability [71, 110]. On the other hand, the charge reversible property improves the electrostatic attraction between the positively charged VCM-HNLCs with the negatively charged bacterial cell surfaces in the acidic environment inside biofilms, which enhances the binding of the NPs and the transport of the antibiotic to the biofilm barrier, hence improving the efficiency of antibacterial agents in eradicating biofilms and inhibiting biofilms formation [111, 112]. These findings suggest that, in addition to the enhanced antibacterial activity against MRSA, VCM-HNLCs may also effectively eliminate bacterial biofilms and modulate biofilm formation.

Recently, bacterial multidrug efflux pumps have garnered considerable attention owing to their potential involvement in clinical AMR [113]. Notably, MRSA has demonstrated the ability to develop efflux-mediated resistance to antibiotics through genetic mutations that diminish bacterial susceptibility to antimicrobial agents [81]. However, the upregulation of bacterial efflux pumps presents a formidable obstacle for antibiotics to efficiently penetrate bacterial cells [114]. Subsequently, the ability of VCM-HNLCs to inhibit MRSA efflux pumps was evaluated. The results indicated VCM-HNLCs treated groups exhibited elevated fluorescence intensity when compared to VCM control groups. This can be attributed to substantial EtBr accumulation within bacterial cells. The presence of TS as an ingredient of the nanocarrier likely contributed to the augmented fluorescence intensity observed in the samples treated with VCM-HNLCs. This augmentation supports the reduction in MIC values and the potentiation of VCM's activity upon incorporation into HNLCs containing TS. Existing literature has reported the promising efflux pump inhibition activity of TS, primarily targeting NorA and NorB, chromosomally encoded efflux pumps found in gram-positive bacteria [46], which may underlie the observed inhibition of efflux pumps in MRSA [42–44]. Therefore, TS may act synergistically with VCM in the formulated VCM-HNLCs to combat resistant *S. aureus* strains and could serve as an adjunct for effective antibiotic therapy, addressing antibacterial resistance attributed to efflux pumps and treating associated conditions such as sepsis.

Excessive production of ROS elicited in response to inflammation precipitates oxidative stress, a significant contributor to the heightened mortality rates observed in sepsis [82]. Therefore, counteracting ROS generated during the inflammatory cascade is paramount in mitigating oxidative stress and ameliorating the deleterious ramifications of sepsis on physiological systems. The radical scavenging ability of the nanosystem was evaluated using the DPPH assay. The findings underscored a concentration-dependent capacity of all tested samples to scavenge DPPH radicals, with maximal antioxidant efficacy recorded at a concentration of 1000 µg/mL. Among the individual excipients, Lys and HA-Lys conjugate exhibited superior scavenging activity compared to TS across all concentrations. This observation may be attributed to the comparatively diminished hydrogen-donating capability of TS compared to the other excipients. Vitamin E derivatives, including TS, have been reported to manifest reduced DPPH scavenging activity relative to pure vitamin E (tocopherol), potentially due to interference from salts in DPPH measurements, consequently diminishing their antioxidant efficacy [115, 116]. Conversely, the heightened antioxidant potential of pure Lys and the synthesized HA-Lys polymer may be ascribed to the presence of free amino groups in Lys units, facilitating electron donation to stabilize and neutralize DPPH radicals [117, 118]. Furthermore, the antioxidant efficacy of VCM-HNLCs may be attributed to the synergistic interplay between TS and HA-Lys, with the HA-Lys conjugate exerting a notable influence over TS. Consequently, the protective role conferred by HA-Lys and its hybrid nanoformulation against DPPH radicals holds promise for attenuating oxidative stress and lipid peroxidation associated with inflammatory cascades and septic conditions [36, 39].

Given that effective treatment of bacterial sepsis relies on eliminating the causative bacteria and modulating the immune response mediated by bacterial toxins such as LPS, we evaluated the anti-inflammatory activity of the formulated VCM-HNLCs by utilizing the HepG2 cell line [55, 68]. To investigate the anti-inflammatory potential of HA-Lys conjugate and VCM-HNLCs formulations against LPS-induced inflammation, we measured cell viability and IL-6 levels using the MTT assay and enzyme-linked immunosorbent assay (ELISA), respectively. The MTT assay revealed that treatment with HA-Lys and VCM-HNLCs significantly improved cell viability in LPS-stimulated cell lines compared to the untreated group. These results indicate that HA-Lys and VCM-HNLCs possess the capability to protect cells from LPS-induced damage. This protective effect may be attributed to the antioxidant properties of lysine, which facilitates the scavenging of reactive oxygen species (ROS) and mitigates oxidative stress, thereby alleviating cellular injury [36, 39]. Additionally, HA has been reported to provide protection against LPS-induced cellular damage in previous studies [119–121]. Consequently, the HA-Lys conjugate and its nanoformulation show promise as potential therapeutic agents for preventing cellular damage caused by sepsis.

Sepsis is intricately linked with dysregulated cytokine expression, which is pivotal for modulating the host immunological response [122]. Among these cytokines, interleukin-6 (IL-6) holds particular significance in the pathogenesis of sepsis, notably serving as an early indicator of inflammation [123]. The anti-inflammatory efficacy of the synthesized HA-Lys and VCM-HNLCs), and their impact assessed on the expression of the pro-inflammatory cytokine, IL-6, in LPS-stimulated HepG2 cells. The results indicated that IL-6 levels were significantly reduced after treatment with varying concentrations of HA-Lys or VCM-HNLCs formulations (50 and 100 µg/mL) compared to the group treated solely with LPS (P-value < 0.0001). This observation underscores the capacity of both HA-Lys and the hybrid nanocarrier to modulate LPS-induced IL-6 levels, thereby potentially mitigating tissue damage resulting from an exaggerated immune response. The outcome corroborates the antagonistic action of both HA-Lys and the hybrid NLCs on TLR4, as their augmented anti-inflammatory effects may be ascribed to their robust and competitive binding to TLR4, as demonstrated in vitro through MST investigations conducted in this study. A similar trend of IL-6 reduction was noted in two studies by Farajzadeh et al. (2018) and Cardoso et al. (2022), which unveiled the significant anti-inflammatory properties of HA-based nanoparticles (NPs) and HA-based nanogels against LPS-triggered inflammatory responses, respectively [124, 125]. Consequently, both HA-Lys and VCM-HNLCs emerge as promising candidates for modulating IL-6 levels and curtailing the inflammatory cascade in septic conditions. Further studies are warranted to evaluate the effect of VCM-HNLCs on other inflammatory markers.

For proof of concept, in vivo mice model of systemic infection was employed to determine the efficacy of VCM-HNLCs. Obtained results indicated that treatment with VCM-HNLCs resulted in a 100% survival rate compared to bare VCM, which exhibited a 50% survival rate after 100 h, as illustrated by the Kaplan–Meier curve in Fig. 15A. This mirrors previous reports of survival rates associated with VCM-loaded liposomes [126]. Additionally, in comparison to free VCM, treatment with VCM-HNLCs led to a significant reduction in MRSA burden across multiple organs. The most substantial decrease in CFU count was observed in the blood, followed by the heart, liver, and lungs, respectively. This pronounced reduction in systemic bacteriemia can be attributed to the sustained effect of the nanosystem in the circulation, facilitated by HA, which is recognized for its prolonged circulatory properties [127]. Literature reports suggest that HA is distributed in the heart and metabolized in the liver, potentially explaining the reduced MRSA burden in these organs, specifically upon the treatment with VCM-HNLCs compared to bare VCM [128]. These findings suggest that VCM-HNLCs have the potential to clear systemic MRSA infection more effectively than bare VCM.

## Conclusion

In recent years, there has been a burgeoning interest in developing advanced, multifunctional biomimetic and stimuli-responsive nanocarriers aimed at enhancing antibiotic delivery for combatting bacterial infections and sepsis. In this study, our objective was to fabricate a novel hyaluronic acid-lysine polymer (HA-Lys) for the construction of biomimetic pH-responsive hybrid nanostructured lipid carriers (HNLCs) capable of facilitating the delivery of vancomycin to address bacterial-induced sepsis. The HA-Lys conjugate was successfully synthesized and characterized by FTIR and ^1^H NMR. Subsequently, vancomycin-loaded HNLCs (VCM-HNLCs) were prepared via a hot homogenization and ultrasonication method. Characterization techniques confirmed satisfactory physicochemical properties, biocompatibility and strong targetability to human TLR2 and TLR4 receptors, as demonstrated by MST studies. Furthermore, VCM-HNLCs exhibited a pH-triggered drug release profile, superior pH-sensitive antibacterial and biofilm eradication capabilities, and significant inhibitory effects against MRSA efflux pumps, substantial free radical scavenging capacities, as well as notable cytoprotective and anti-inflammatory effects against LPS-induced inflammation compared to the unencapsulated VCM. These characteristics hold promise for enhancing the anti-virulence efficacy of VCM and improving outcomes in sepsis treatment.

While the study demonstrates promising results for the use of VCM-HNLCs as a potential treatment for bacterial sepsis, further research is needed to confirm their antibacterial activity against a broader range of bacterial strains. Additionally, investigations into the purity, long-term stability, pharmacokinetics, and potential toxicity of VCM-HNLCs are essential for their clinical application. The development of preclinical sepsis models that closely resemble human septic conditions is crucial. Additionally, evaluating the effects of VCM-HNLCs on various tissues through pathohistological investigations, assessing their in vivo anti-inflammatory effects, and exploring the biological behavior and mechanistic molecular pharmacodynamics of the system will be essential for advancing their clinical potential. The outcomes of this research lay the foundation for developing novel nanoplatforms to manage bacterial sepsis and combat multidrug-resistant bacteria, contributing to the expansion of hybrid nanostructured lipid carriers with antivirulence properties that integrate biomimetic and stimuli-responsive strategies to enhance antibiotic therapy against sepsis-inducing pathogens.

## Supplementary Information

Supplementary Material 1

## Data Availability

All data supporting the findings of this study are included in the main article and supplementary materials. Raw data can be provided upon request by contacting the corresponding authors.
