# Supplementary material for: Multifunctional hyaluronic acid-based biomimetic/pH-responsive hybrid nanostructured lipid carriers for treating bacterial sepsis
Source: J Biomed Sci. 2025 Feb 11;32:19. doi: 10.1186/s12929-024-01114-6 (PMC11812216; doi:10.1186/s12929-024-01114-6)
Supplement: Supplementary file 1 — Supplementary Material 1 [file 12929_2024_1114_MOESM1_ESM.docx]

**Multifunctional hyaluronic acid-based Biomimetic/pH-responsive hybrid nanostructured lipid carriers for Treating Bacterial Sepsis**

Supplementary Materials

Eman Elhassan ^a^, Calvin A. Omolo ^a,c^ **^*^**, Mohammed A. Gafar ^a,b^, Eman A. Ismail ^a,^ Usri H. Ibrahim ^d^, Rene Khan ^e^, Mathieu Lesouhaitier ^f^, Paul Kubes ^f^, Thirumala Govender ^a^ **^*^**.

^a^ Discipline of Pharmaceutical Sciences, College of Health Sciences, University of KwaZulu-Natal, Private Bag X54001, Durban, South Africa.

^b^ Department of Pharmaceutics, Faculty of Pharmacy, University of Khartoum, Sudan.

^c^ Department of Pharmaceutics and Pharmacy Practice, School of Pharmacy and Health Sciences, United States International University-Africa, P. O. Box 14634-00800, Nairobi, Kenya.

^d^ Discipline of Human Physiology, School of Laboratory Medicine and Medical Sciences, College of Health Sciences, University of KwaZulu-Natal, Durban, South Africa.

^e^ Discipline of Medical Biochemistry, School of Laboratory Medicine and Medical Science, University of KwaZulu-Natal, Durban, South Africa.

^f^ Department of Physiology and Pharmacology, Cumming School of Medicine, University of Calgary, Calgary, AL, Canada

^*^ Corresponding authors: [comolo@usiu.ac.ke](mailto:comolo@usiu.ac.ke), [govenderth@ukzn.ac.za](mailto:govenderth@ukzn.ac.za).

**Keywords:** Biomimetic; pH-responsive; Hyaluronic acid-lysine; Hybrid nanocarriers; Bacterial Sepsis; Antibiotic delivery.


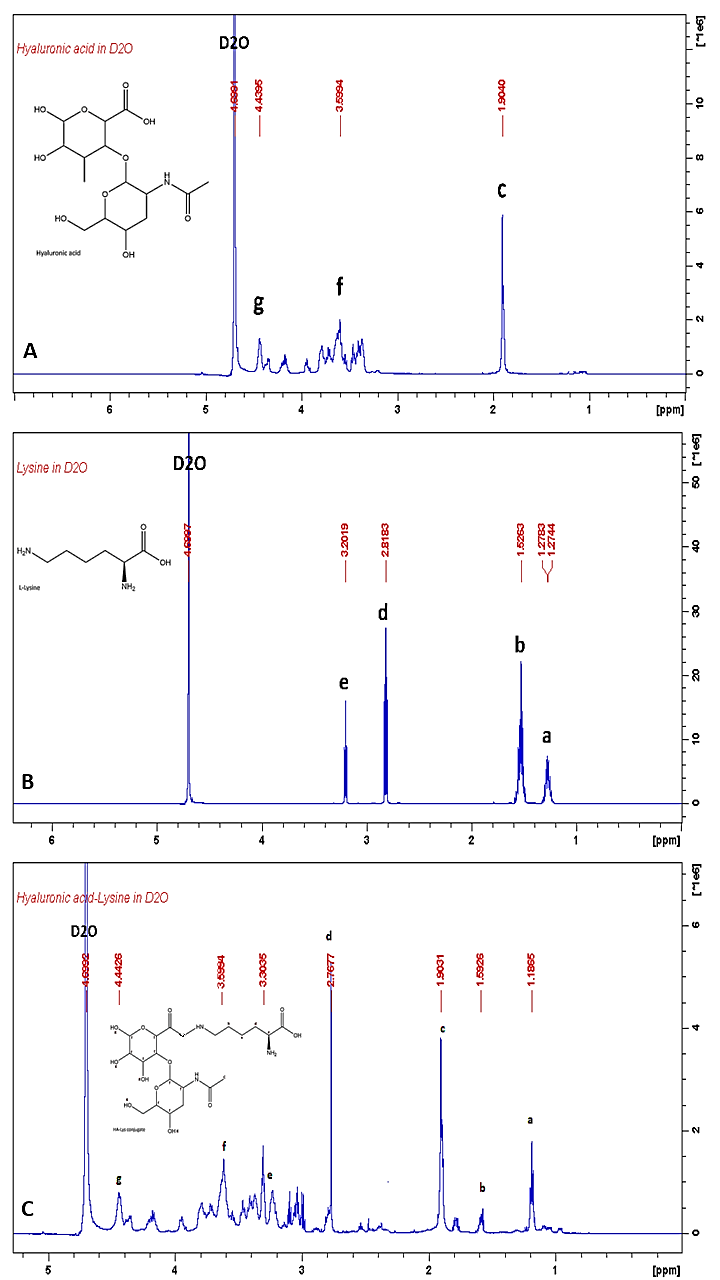


**Figure 1. ^1^**H NMR characterization of (**A**) HA, (**B**) Lys and (**C**) HA-Lys conjugate in D_2_O.

(*HA=Hyaluronic acid, Lys=L-Lysine, HA-Lys= Hyaluronic acid-lysine conjugate)


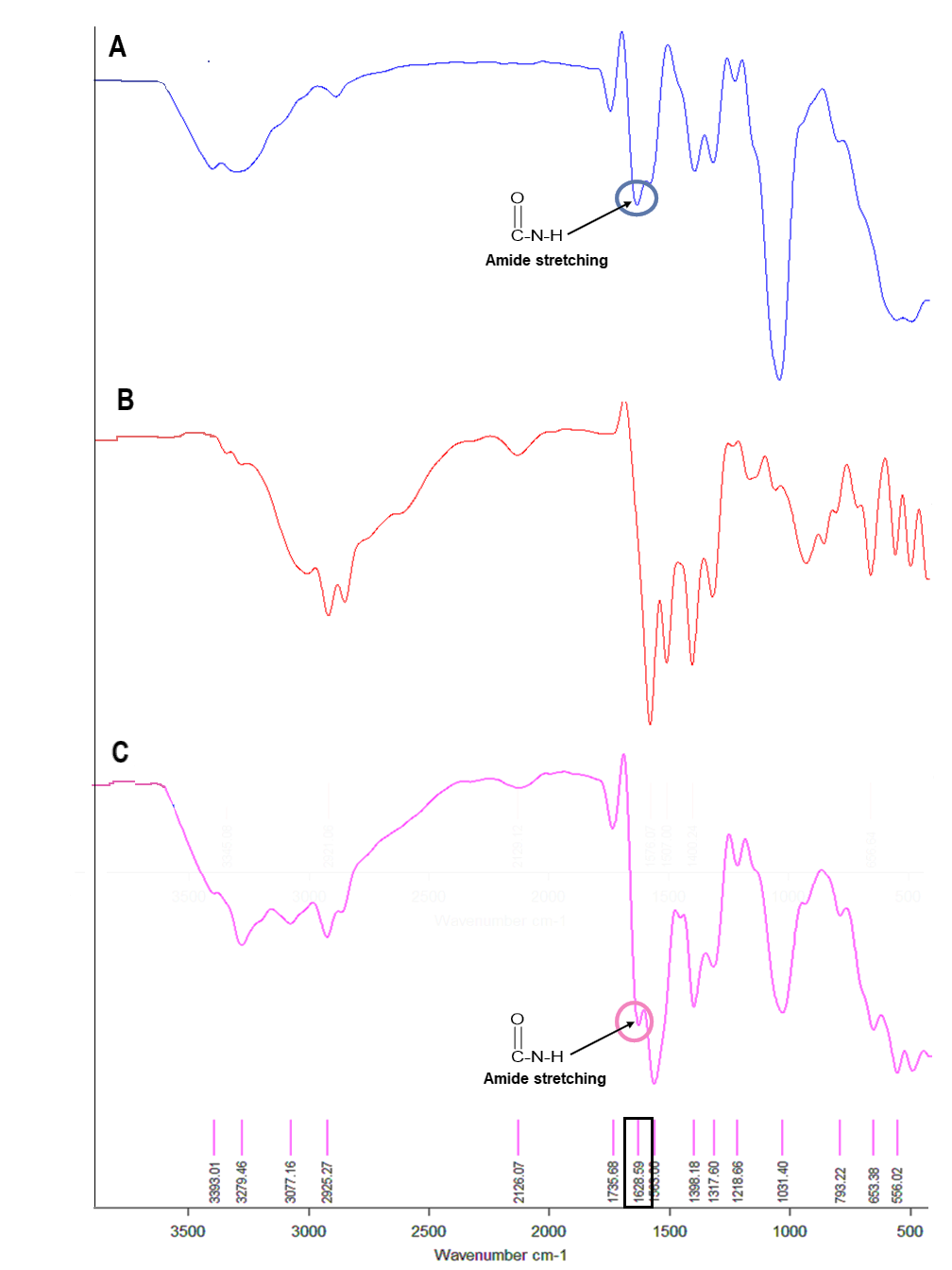


**Figure 2.** FT-IR spectrum of (**A**) HA, (**B**) Lys and (**C**) HA-Lys conjugate. (*HA=Hyaluronic acid, Lys=L-Lysine, HA-Lys= Hyaluronic acid-lysine conjugate)

**Figure 3**. Vancomycin calibration curve

**Table 1.** The FIC Index

| **FIC index** | **Interpretation** |
| --- | --- |
| ≤0.5 | Synergy |
| >0.5 to 1 | Additive |
| >1 to <2 | Indifference |
| ≥2 | Antagonism |

**Table 2.** Shows the effect of surfactant type on size, PDI and ZP of different HNLCs formulations (blank HNLCs) Data are presented as mean ± SD (n = 3).

| Surfactant | Size(nm) | PDI | ZP (mV) |
| --- | --- | --- | --- |
| Span 80 | 3162 ± 533.300 | 1.000 ± 0.000 | -5.55 ± 0.220 |
| PEG 400 | 980.7 ± 86.750 | 0.629 ± 0.060 | -5.74 ± 0.460 |
| PVP | 510.1 ± 6.440 | 0.136 ± 0.030 | -5.57 ± 1.380 |
| PLX (P 188) | 2082 ± 54.640 | 1.000 ± 0.000 | -6.22 ± 0.623 |
| Tween 80 | 65.44 ± 0.438 | 0.255 ± 0.010 | -4.07 ± 0.516 |


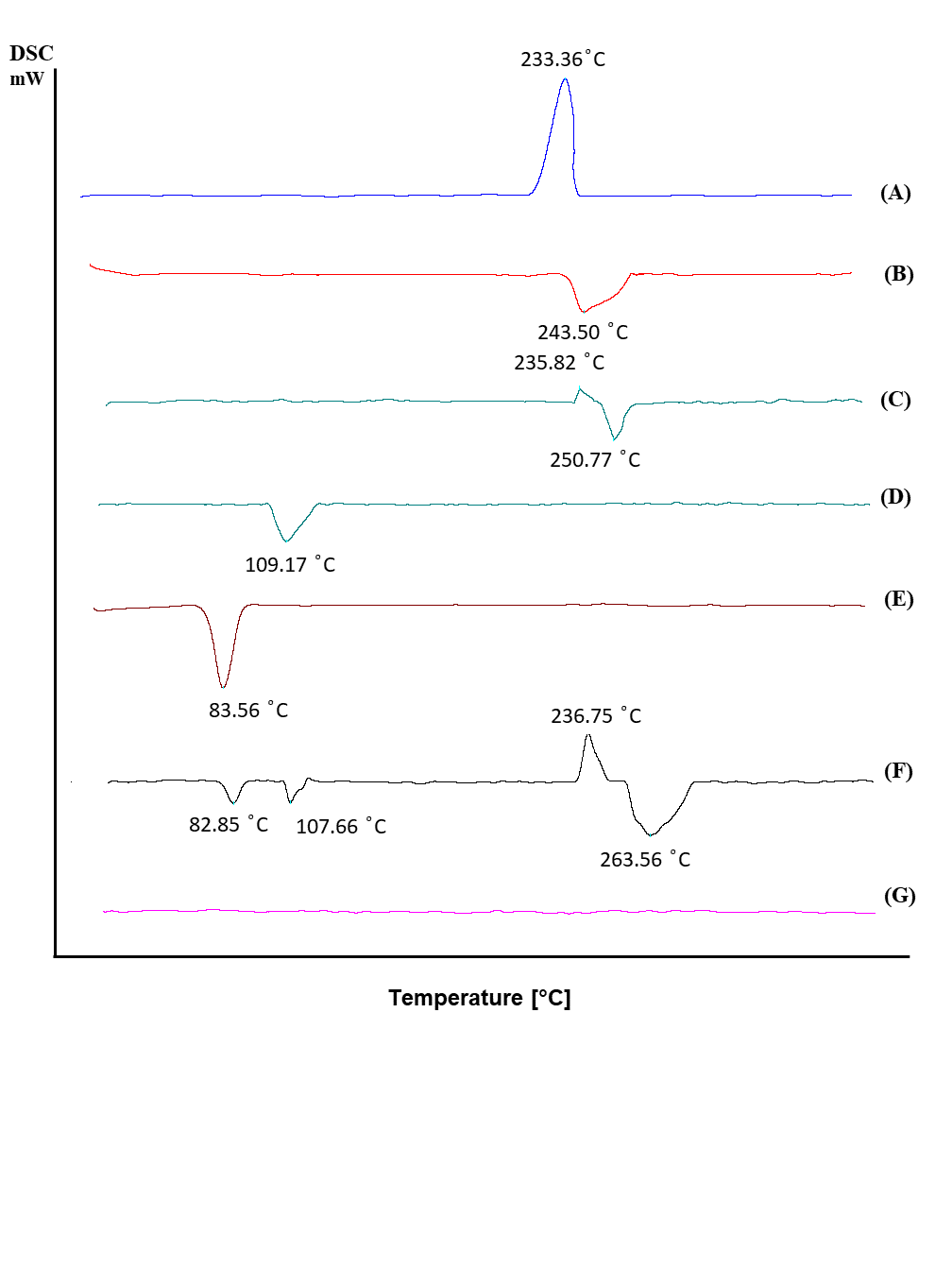


**Figure 4**. DSC thermograms of (**A**) Hyaluronic acid (HA); (**B**) _L_-Lysine (Lys); (**C**) HA-Lys conjugate; (**D**) Vancomycin; (**E**) Tocopherol succinate; (**F**) physical mixture; (**G**) lyophilized VCM-HNLCs formulation.

**Table 3**. Hemolysis (%) for different concentrations of VCM-HNLCs, PBS (pH 7.4) and distilled water (D.W). Data are presented as mean ± SD (n = 3).

| **VCM-HNLCs (mg/mL)** | **Hemolysis %** |
| --- | --- |
| 0.5 | 0.397 ± 0.312 |
| 0.1 | 0.193 ± 0.037 |
| 0.2 | 0.332 ± 0.112 |
| 0.3 | 0.446 ± 0.280 |
| 0.4 | 0.382 ± 0.182 |
| 0.5 | 0.631 ± 0.770 |
| PBS | 0 |
| D.W | 100 |

**Table 4.** The results of particle size (nm), PDI and ZP (mV) of VCM-HNLCs under different storage durations. Data are presented as mean ± SD (n = 3).

| **Day** | **RT** | | | **4° C** | | |
| --- | --- | --- | --- | --- | --- | --- |
|  | **Size (nm)** | **PDI** | **ZP (mV)** | **Size (nm)** | **PDI** | **ZP (mV)** |
| **0** | 110.7±1.693 | 0.113±0.022 | -2.92±0.214 | 110.7±1.696 | 0.113± 0.020 | -2.92± 0.212 |
| **30** | 110.3±1.161 | 0.093±0.010 | -4.04±1.171 | 114.0±1.235 | 0.103±0.026 | -3.28±0.981 |
| **60** | 110.8±1.210 | 0.093±0.024 | -4.42±0.436 | 115.8±1.902 | 0.106±0.038 | -4.37±1.480 |
| **90** | 113.3±1.183 | 0.108±0.016 | -4.72±0.864 | 115.6±1.284 | 0.103±0.010 | -4.10±0.690 |

******RT****= room temperature*
